# Single-cell phenotype-associated subpopulation identification via transfer foundation model and statistical ensemble learning

**DOI:** 10.1186/s12915-026-02613-8

**Published:** 2026-04-29

**Authors:** Yuming Zhao, Xiaonan Pan, Zeyu Luo, Qiaoming Liu

**Affiliations:** 1 College of Computer and Control Engineering, Northeast Forestry University, Harbin, 150040 China; 2 School of Artificial Intelligence, Henan University, Zhengzhou, 450000 China

**Keywords:** Pre-trained foundation model (PFM), Transfer learning, Single-cell RNA sequencing, Cell subpopulation identification, Data integration

## Abstract

**Background:**

Single-cell RNA sequencing (scRNA-seq) enables the characterization of cell types, states, and lineages within heterogeneous tissues, thereby providing unprecedented opportunities to dissect cellular heterogeneity. However, single-cell data alone cannot directly establish cell-phenotype relationships, which pose major challenges in linking cellular heterogeneity to complex traits and disease outcomes.

**Results:**

Here, we introduce scPASI, which integrates single-cell and bulk-level information to uncover phenotype-associated cell subpopulations. scPASI combines a pre-trained foundation model (PFM) with a residual variational autoencoder (Res-VAE) to extract feature embeddings of cells and samples. Cell clusters are calculated using the Leiden algorithm, after which phenotype associations are inferred based on regression coefficients derived from LASSO and sparse group LASSO (SGL) models. This design enables scPASI to stratify cells into four subpopulations with different levels of phenotype association: strongly positive (SP), weakly positive (WP), strongly negative (SN), and weakly negative (WN) groups. Furthermore, scPASI characterizes phenotype-relevant genes within subpopulations and provides insights into the relationship between cellular heterogeneity and bulk phenotypes. Extensive evaluations across diverse datasets show that scPASI outperforms existing methods and generalizes well across multiple phenotype settings, including tumor status, genetic mutations, and clinical prognosis. Biological analyses demonstrate that signature genes derived from the identified subpopulations can distinguish tumor cells, genetic alterations, and survival outcomes.

**Conclusions:**

By bridging single-cell transcriptomics with phenotype information, scPASI can uncover biologically meaningful cell-phenotype associations underlying tumor biology, enabling the identification of disease-relevant subpopulations and providing a framework for potential therapeutic targeting.

**Supplementary Information:**

The online version contains supplementary material available at 10.1186/s12915-026-02613-8.

## Background

Cancers are driven by complex interactions among diverse cellular populations, heterogeneous gene expression programs, and dynamic remodeling of the tumor microenvironment [1, 2]. Bulk RNA sequencing (RNA-seq) techniques generate high-quality gene expression profiles together with extensive clinical annotations, as exemplified by resources such as The Cancer Genome Atlas (TCGA) [3] and the Genotype-Tissue Expression (GTEx) project [4], making them well-suited for modeling patient-level phenotypes. However, bulk RNA-seq measures averaged gene expression across tissue samples, which mask the functional states and phenotype-driving mechanisms of critical cellular subpopulations within individual patients [5]. The advent of single-cell RNA sequencing (scRNA-seq) has revolutionized disease research by enabling high-resolution characterization of cellular heterogeneity, reconstruction of developmental trajectories, and identification of regulatory cells within complex tissues [6–10]. Despite its ability to capture cell-type-specific molecular signatures, scRNA-seq data lack direct correspondence to patient-level clinical phenotypes, such as survival outcomes, disease recurrence, or treatment responses [11, 12]. Moreover, given that changes in the composition and functional states of specific cell subsets critically influence disease initiation, progression, and clinical outcomes [13, 14], identifying these subsets requires phenotype information derived from bulk transcriptomic datasets. Therefore, integrative analytical frameworks that combine scRNA-seq data with phenotype-annotated bulk RNA-seq data are essential, as they enable the systematic identification of phenotype-associated cellular subpopulations and reveal their contributions to disease pathogenesis and clinical prognosis [15–17].

Concurrently, the emergence of pre-trained foundation models (PFMs), which have been rapidly adapted for bioinformatics applications [18–20], has opened new opportunities for modeling high-dimensional omics data and enabling knowledge transfer across datasets. By capturing latent semantic structures within complex transcriptomic datasets, PFMs facilitate the identification of phenotype-associated features and improve the modeling of intricate cellular states, thereby enhancing the prediction of cell states, disease trajectories, and clinical outcomes [21]. Notably, transcriptomics-oriented PFMs such as Geneformer [22] and scFoundation [23], pretrained on millions of single-cell transcriptomes, generate transferable representations that substantially advance single-cell transcriptomic analysis and broaden its applications to disease modeling. In parallel, variational autoencoders (VAEs) have emerged as powerful tools for learning latent representations of scRNA-seq data and correcting batch effects. Models such as scVI [24] and scVAE [25] construct unified latent spaces that denoise high-dimensional input data while preserving key biological signals. These latent representations provide a robust foundation for integrating scRNA-seq data with bulk RNA-seq profiles, enabling cross-level biological discovery.

Various methodologies have been developed for integrative analysis of bulk and single-cell RNA-seq data. Deconvolution techniques, including CIBERSORTx [26] and MuSiC [27], leverage scRNA-seq data as reference profiles to infer cell-type proportions in bulk samples. However, these approaches rely on predefined cell-type annotations, which limit their ability to discover novel functional cell subpopulations. More recent methods such as Scissor [28] and scAB [29] employ graph-based frameworks and regression-based modeling to identify phenotype-associated cellular subpopulations without requiring prior cell-type labels, representing an important advancement in phenotype-driven single-cell analysis.

However, existing approaches often rely on raw gene expression profiles or linearly reduced features, which may fail to capture complex contextual relationships among genes. In contrast, PFMs excel at this task by learning high-level representations that encode gene–gene interactions and contextual biological information [30]. Additionally, many current strategies simplify phenotype association analysis into binary classifications, categorizing cells as phenotype-positive or phenotype-negative based solely on the sign of regression coefficients. Such binary categorization overlooks the graded nature of phenotype associations among heterogeneous cell populations. In complex disease contexts such as cancer, certain cells may exhibit strong and consistent associations with adverse phenotypes, whereas others may show weaker or context-dependent effects. The inability of existing approaches to distinguish these patterns may obscure critical biological insights embedded within tumor heterogeneity.

**Fig. 1:**
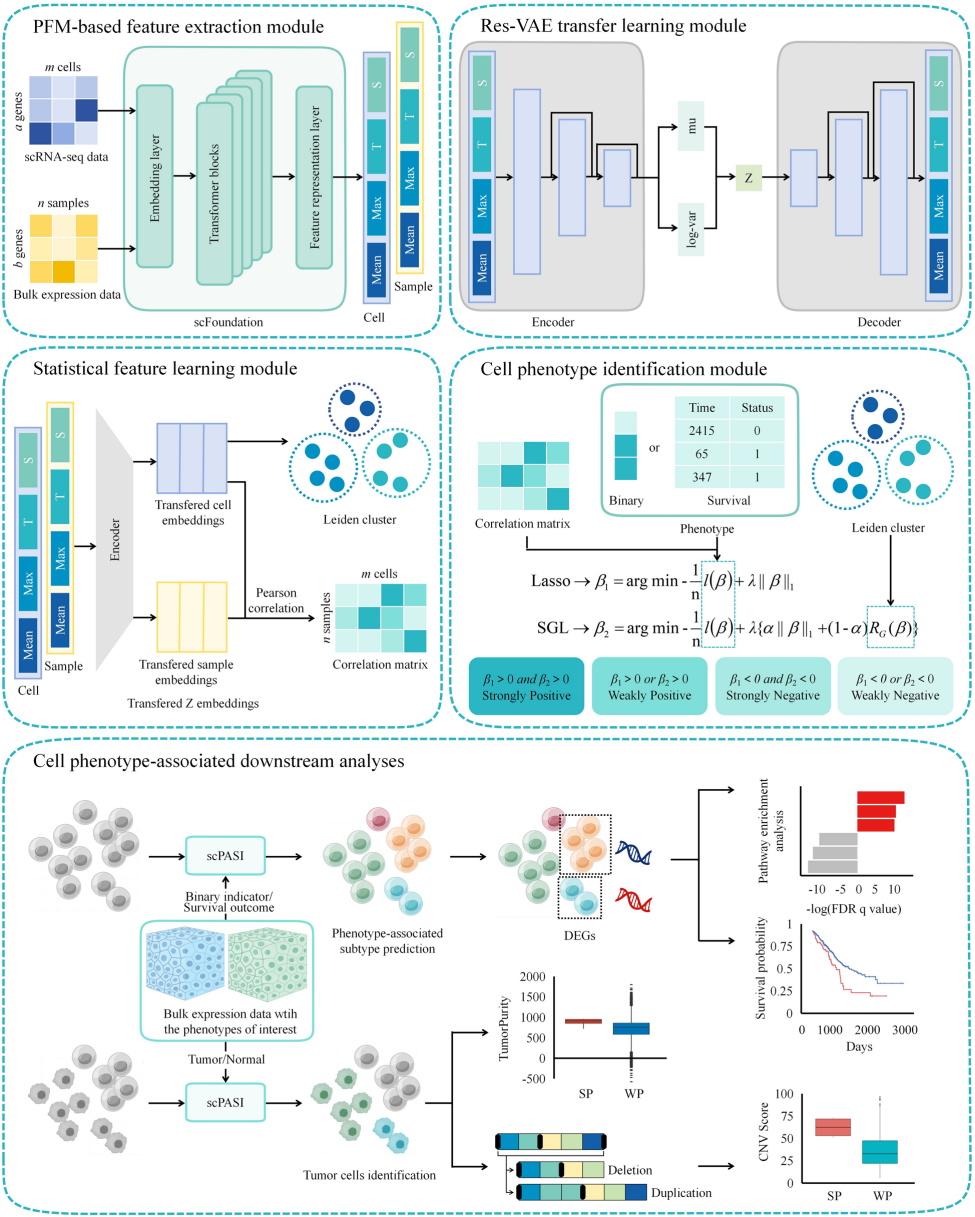
The scPASI framework comprises four sub-modules. (i) The PFM-based feature extraction module. Raw scRNA-seq and bulk RNA-seq data are processed using a pre-trained scFoundation model to extract feature embeddings representative of single cells and bulk samples. (ii) The Res-VAE transfer learning module. scATD was trained on the PanglaoDB dataset using tenfold cross-validation, and its pretrained weights were used to initialize the Res-VAE to obtain modality-specific embeddings via transfer learning. (iii) The statistical feature learning module. The feature embeddings generated by scFoundation are processed through the Res-VAE encoder to produce transferred embeddings, which are then utilized to compute a Pearson correlation matrix for capturing cell-to-sample relationships. The Leiden algorithm is employed to generate prior cell grouping labels, which are then used as input for the SGL regression model. (iv) The cell phenotype identification module. An ensemble regression approach combining LASSO and SGL models calculates regression coefficients for each cell, enabling their stratification into four cell subpopulations based on association strength with the phenotype of interest: SP, WP, SN, and WN groups. The identified subpopulations are subjected to downstream analyses, including: tumor cell characterization through evaluation of tumor malignancy and purity to quantify biological heterogeneity between subpopulations; and phenotype-driven discovery via differential gene expression analysis, as well as pathway enrichment and survival analysis based on subpopulation-specific gene signatures

To address these challenges, we propose scPASI, a computational framework for Single-cell Phenotype-associated Subpopulation Identification (Fig. 1). scPASI extracts features from transcriptomic data and performs statistical analysis on regression coefficients to stratify cells into four phenotype-associated subpopulations: Strongly Positive (SP), Weakly Positive (WP), Strongly Negative (SN), and Weakly Negative (WN) groups. To systematically capture phenotype-associated cellular heterogeneity, scPASI integrates representation learning, nonlinear latent refinement, and phenotype-guided regression into a unified framework. Specifically, scPASI combines the pre-trained foundation model scFoundation with a Residual Variational Autoencoder (Res-VAE) to facilitate feature extraction and identify phenotype-associated cell subpopulations across scRNA-seq and bulk RNA-seq datasets. The scFoundation model generates biologically informative cell-level embeddings from transcriptomic profiles. These embeddings are subsequently refined and compressed through the Res-VAE encoder to obtain lower-dimensional latent representations, which reduce redundancy and enhance downstream similarity estimation. Based on these representations and phenotype information, scPASI applies the Least Absolute Shrinkage and Selection Operator (LASSO) [31] and Sparse Group LASSO (SGL) [32] regression models to infer associations between cell subpopulations and phenotypes. Specifically, LASSO and SGL perform phenotype-guided regression on the sample-cell similarity matrix, introducing sparsity at both individual and group levels to identify phenotype-associated cell subpopulations from heterogeneous transcriptomic datasets in an interpretable manner. This integrated design enables scPASI to effectively identify phenotype-related cell subpopulations and achieve robust and accurate identification across heterogeneous datasets. We benchmark scPASI against representative methods across diverse disease datasets encompassing multiple phenotype settings, including tumor versus normal status, overall survival (OS) information, TP53 mutant versus wild-type, and patient vital status. Across these datasets, scPASI achieves comparable or superior performance, as reflected by higher AUC and C-index values, and demonstrates greater stability and accuracy in phenotype-associated cell subpopulation identification. Through further biological analyses of the cell subpopulations identified by scPASI across different phenotypes in diverse disease datasets, scPASI offers enhanced biological interpretability, enabling more meaningful insights into cellular heterogeneity and phenotype-cell associations. By enabling precise and fine-grained identification of phenotype-associated cellular subpopulations, scPASI provides a versatile analytical framework with broad applicability to studying disease mechanisms and facilitates the discovery of potential therapeutic targets.

## Results

### scPASI enables accurate identification of phenotype-associated cell subpopulations

Identifying phenotype-associated cell subpopulations is critical for deciphering complex biological processes and understanding disease mechanisms. scPASI integrates phenotypic information with transferred embeddings derived from the PFM and Res-VAE modules in scRNA-seq and bulk RNA-seq data, enabling robust identification of cell subpopulations linked to specific phenotypes. A series of benchmark analyses was conducted to systematically evaluate scPASI’s performance in phenotype-associated subpopulation identification. The framework integrates PFM and Res-VAE for feature extraction, Leiden clustering (with a default *resolution* parameter of 0.5), and the SGL with LASSO (SGL_LASSO) regression module. Leveraging the NSCLC and COAD datasets with ground-truth tumor labels, the full scPASI framework was compared against three existing methods (Scissor [28], scAB [29], and scIdentifier [33]) based primarily on the number of correctly detected tumor cells, with a detailed method-level comparison provided in the supplementary file (Additional file 1, Table S1). Subsequently, six disease datasets with phenotype information, including tumor versus normal (NSCLC, COAD), overall survival (BRCA, LIHC, LUAD), TP53 mutant versus wild-type (LUAD, HNSC), and binary survival outcome (NSCLC), were analyzed using the area under the curve (AUC) and the concordance index (C-index) as evaluation metrics.

The performance of scPASI was evaluated in identifying cell subpopulations by comparing it against existing methods (Scissor, scAB, and scIdentifier) based on the number of accurately identified tumor cells. In the NSCLC dataset, scPASI identified 173 tumor cells (subdivided into SP and WP groups), which is more than scIdentifier (137 cells); whereas in the COAD dataset, scPASI accurately identified 390 tumor cells, a performance comparable to Scissor (400 cells) and higher than scAB (312 cells) and scIdentifier, which failed to identify any tumor cells (Table 1). Although Scissor yielded higher counts of correctly identified tumor cells, it cannot identify the direction (positive or negative) and the strength (strong or weak) of the tumor phenotype for each cell. Because our goal is robust four-class stratification with consistent phenotype direction and stable effect sizes, we emphasize directional consistency and stability in addition to the count-based metric. In contrast, according to the sign and magnitude of the inferred regression coefficients, scPASI stratified cells into four subpopulation types, including strongly positive (SP), weakly positive (WP), strongly negative (SN), and weakly negative (WN), highlighting its unique capacity to capture more precise cell phenotypes.

**Table 1:** Comparison of scPASI and existing methods on the number of correctly identified tumor cells

Dataset	Method	Number of correctly identified tumor cells
NSCLC	scPASI	173
	scIdentifier	137
	scAB	336
	Scissor	522
COAD	scPASI	390
	scIdentifier	0
	scAB	312
	Scissor	400

To determine whether the WP category reflects biologically intermediate states rather than statistical instability arising from model disagreement, additional robustness analyses were conducted. Two datasets, LUAD_TP53 and COAD_tumors, were selected for subsampling-based stability evaluation. In each iteration, positively associated cells were retained to preserve the underlying phenotype structure. In contrast, negatively associated cells were randomly downsampled to introduce controlled perturbations (see the “Datasets” section for details). The regression models were then re-fitted, and cell subpopulation labels (SP/WP/SN/WN) were reassigned for each resampled dataset.

Stability analysis demonstrated that WP cells remained highly consistent across increasing perturbation levels. In the LUAD_TP53 dataset, no WP cells transitioned to negative categories across all downsampling ratios (10%–90%). Transitions from WP to SP were observed at low frequencies, ranging from 0.61% to 5.52%, with no monotonic increase under stronger perturbation (Table 2). Similarly, in the COAD_tumors dataset, WP classification was remarkably stable. No WP-to-negative transitions were observed. Only minimal WP-to-SP transitions occurred, with rates below 0.28% even at the highest perturbation level (Additional file 2, Table S2). Overall, the extremely low transition rates and absence of polarity reversal (positive to negative) indicate that WP cells do not arise from random disagreement between regression models. Instead, they represent stable intermediate phenotype-associated states that are robust to substantial sampling perturbation.

**Table 2:** Stability analysis of WP cells under progressive downsampling (LUAD_TP53)

Downsampling ratio of negative cells	WP → Negative	Flip rate (%)	WP → SP	Flip rate (%)
10% removed	0	0.0000	3	1.8405
20% removed	0	0.0000	4	2.4540
30% removed	0	0.0000	7	4.2945
40% removed	0	0.0000	9	5.5215
50% removed	0	0.0000	5	3.0675
60% removed	0	0.0000	1	0.6135
70% removed	0	0.0000	3	1.8405
80% removed	0	0.0000	4	2.4540
90% removed	0	0.0000	6	3.6810

**Fig. 2:**
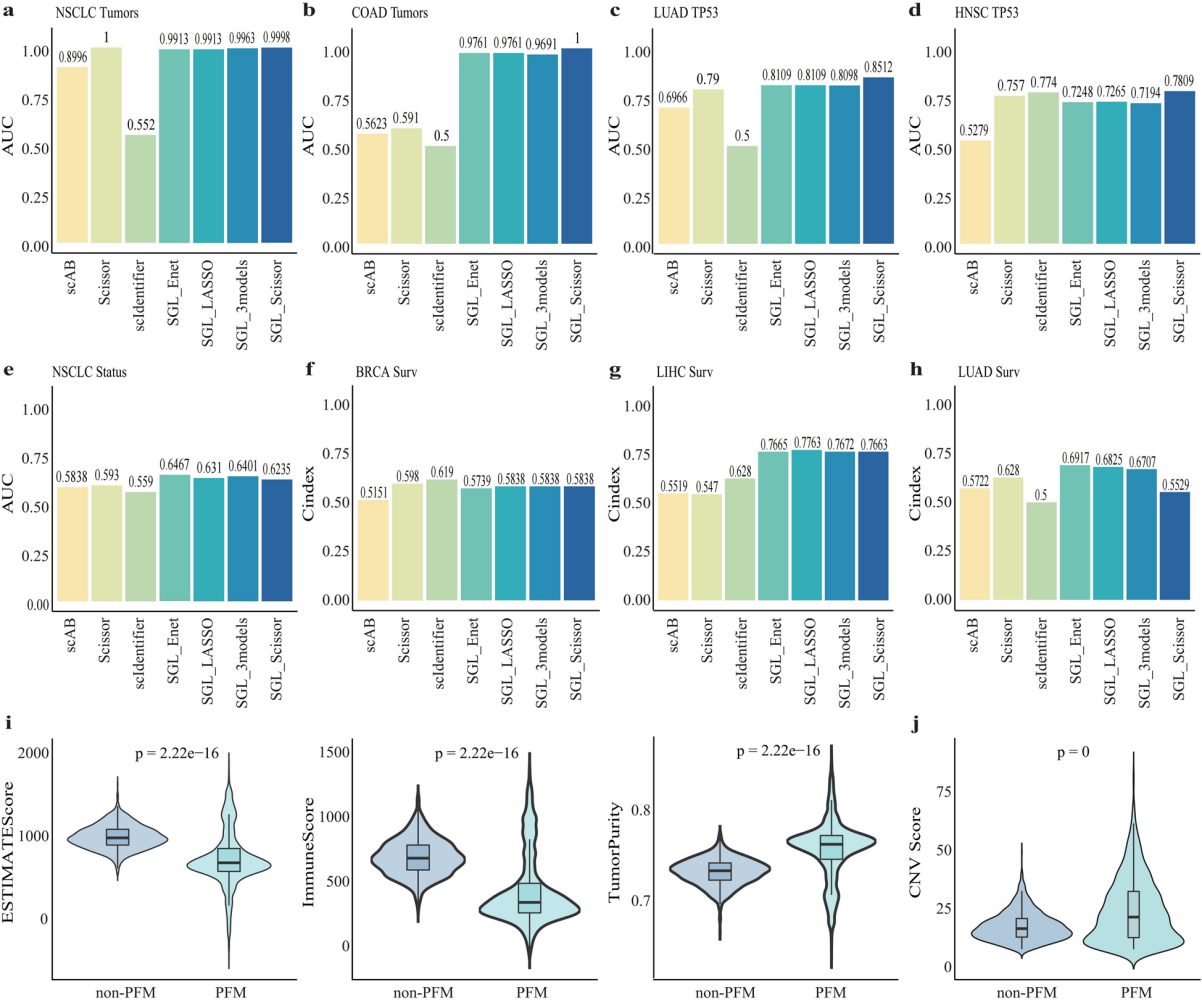
Performance evaluation of scPASI across multiple datasets and phenotypes. **a**–**e** AUC comparisons using five datasets with various phenotypes: NSCLC (tumor vs. normal), COAD (tumor vs. normal), LUAD (TP53 mutant vs. wild-type), HNSC (TP53 mutant vs. wild-type), and NSCLC (alive vs. deceased status). The bar plots show the AUC values on scPASI compared with different regression model combinations and existing methods. **f**–**h** C-index comparisons using three datasets with overall survival: BRCA, LIHC, and LUAD. The bar plots show the C-index values on scPASI compared with different regression model combinations and existing methods. **i** Comparison of scPASI with and without PFM in terms of ESTIMATE scores, immune scores, and tumor purity scores for cells identified in the NSCLC dataset (tumor vs. normal phenotype). **j** Comparison of scPASI with and without PFM in terms of CNV scores for cells identified in the same NSCLC dataset

Additionally, the accuracy of phenotype-associated cell subpopulation identification was evaluated to assess scPASI’s generalizability against other methods across eight disease-phenotype datasets including NSCLC (tumor vs. normal), COAD (tumor vs. normal), LUAD (TP53 mutant vs. wild-type), HNSC (TP53 mutant vs. wild-type), NSCLC (alive vs. deceased status), BRCA (overall survival), LIHC (overall survival), and LUAD (overall survival). scPASI consistently achieves comparable predictive accuracy and concordance (AUC and C-index, Fig. 2a–h) compared to other methods and alternative regression model combinations.

In summary, these results demonstrate that scPASI exhibits robust and superior performance over existing methods across a broad spectrum of phenotypic associations, including tumor status, gene mutation, overall survival, and binary survival outcomes. Importantly, the WP subpopulation stratification is highly stable under substantial negative-cell downsampling, indicating that WP cells reflect biologically intermediate states rather than statistical noise. Moreover, by integrating LASSO and SGL regression models, scPASI enables a refined four-class classification (SP, WP, SN, WN), providing fine-grained phenotype classification and interpretability of phenotype-associated cellular heterogeneity that cannot be captured by single-model or binary classification approaches. These features collectively support the reliability of scPASI in dissecting complex disease-associated cellular landscapes.

### Evaluation of key scPASI sub-modules for cell subpopulation identification

Within the scPASI framework, the scFoundation model is incorporated to extract feature embeddings from scRNA-seq and bulk RNA-seq data, followed by refinement through a Res-VAE module for dimension alignment. Experiments on the NSCLC and COAD datasets were performed to systematically evaluate the contributions of several key components: PFM versus non-PFM features, alternative clustering algorithms, varying *resolution* parameters, and different regression model combinations. Additionally, ablation analyses of the regression models, where LASSO and SGL are applied individually, were conducted across eight disease-phenotype association datasets to assess their specific contributions to performance and subpopulation stratification.

To specifically assess the contribution of PFM-based features, the strongly positive (SP) and weakly positive (WP) cells identified by scPASI with PFM in the NSCLC and COAD datasets were systematically analyzed regarding tumor malignancy and purity. Tumor malignancy was evaluated based on copy number variation (CNV) scores, which reflect the extent of genomic instability and indicate aggressive tumor behavior. In contrast, tumor purity was calculated using the *ESTIMATE* package. In the NSCLC dataset, tumor cells identified by scPASI with PFM exhibited significantly higher tumor purity scores and lower immune scores than those identified without PFM (Fig. 2i). CNV analysis further revealed that tumor cells identified by scPASI with PFM exhibited higher CNV scores than those identified without, indicating more pronounced malignant genomic alterations (Fig. 2j) [34]. Similar trends were observed in the COAD dataset, confirming that including PFM enhances the biological accuracy of tumor cell identification (Additional file 5, Figure S1). These results demonstrate that integrating PFM into scPASI considerably improves its sensitivity to tumor-specific transcriptional signatures, enabling more precise discrimination of tumor cells. By strengthening feature representation across modalities, the PFM component enhances the biological relevance of phenotype-associated subpopulation identification.

Within the SGL regression model, the definition of prior cell grouping labels constitutes an essential prerequisite, and choosing the clustering algorithm and its *resolution* parameter is critical for accurately identifying phenotype-associated subpopulations. To systematically justify the use of the Leiden algorithm for generating single-cell cluster labels in scPASI and to determine an optimal *resolution* parameter, seven clustering algorithms (Leiden, Louvain [35], K-means [36], Leiden-CPM, Leiden-Significance, Leiden-Surprise, and FLPA [37]) were evaluated across *resolution* values ranging from 0.1 to 0.9 in increments of 0.1. This benchmarking was performed on NSCLC and COAD datasets containing ground-truth tumor cell labels, enabling direct assessment of each algorithm’s performance in identifying tumor-related subpopulations. Results showed that both the Leiden and Louvain algorithms consistently identified more ground-truth tumor cells than other cluster algorithms (Table 3). Although K-Means clusters were determined using the elbow method to select the number of clusters, its performance varied considerably between datasets and was less stable compared to graph-based methods, making it a less reliable choice for robust identification of phenotype-associated subpopulations. However, the Louvain algorithm exhibited considerable fluctuations in tumor cell counts across the tested *resolution* settings (0.1 to 0.9, in increments of 0.1) and aberrant clustering behavior at high *resolution* values. In contrast, Leiden produced more stable and biologically plausible tumor cell distributions, with the *resolution* interval of 0.4–0.6 showing firm agreement with clinical annotations (Additional file 3, Table S3). Based on these observations, Leiden is preferred because it offers a better balance between biological plausibility, stability, and consistency across datasets.

**Table 3:** Comparison of cell grouping labels from different clustering algorithms on the number of correctly identified tumor cells

Dataset	Cluster algorithm	Number of correctly identified tumor cells
NSCLC	Leiden	173
	Louvain	161
	KMeans	1142
	Leiden-CPM	5
	Leiden-Significance	5
	Leiden-Surprise	6
	FLPA	5
COAD	Leiden	390
	Louvain	439
	KMeans	171
	Leiden-CPM	16
	Leiden-Significance	12
	Leiden-Surprise	12
	FLPA	23

The NSCLC and COAD datasets, which include experimentally validated ground-truth tumor labels, were used to compare the SGL with LASSO (SGL_LASSO) combination against other regression model combinations: SGL with Elastic Net (SGL_Enet), SGL with Scissor (SGL_Scissor), and SGL integrating LASSO and group LASSO (SGL_3models). Performance was assessed using the area under the curve (AUC), the number of correctly identified tumor cells, and the false positive rate (FPR). The SGL_LASSO model achieved high accuracy in both datasets, with AUC values of 0.9926 in NSCLC and 0.9681 in COAD using Leiden clustering at a *resolution* of 0.6. It also demonstrated competitive performance against other models (Additional file 4, Table S4) and exhibited the lowest FPR across both datasets (Table 4). Furthermore, in BRCA, LIHC, and LUAD datasets incorporating overall survival (OS), SGL_LASSO consistently showed high concordance indices, indicating robust prognostic predictive ability (Fig. 2f–h). In contrast, alternative combinations underperformed in several aspects: SGL_3models did not significantly increase true tumor cell detection, SGL_Scissor yielded less consistent survival predictions, and SGL_Enet was associated with elevated FPRs. These results affirm SGL_LASSO as the regression model of choice within the scPASI framework, balancing high predictive accuracy with biological relevance.

**Table 4:** Comparison of different regression model combinations on false positive rate (FPR)

Dataset	Regression model combination	FPR
NSCLC	SGL_LASSO	0.033800
	SGL_Enet	0.034200
	SGL_3models	0.044500
	SGL_Scissor	0.056400
COAD	SGL_LASSO	0.204144
	SGL_Enet	0.205363
	SGL_3models	0.240701
	SGL_Scissor	0.295551

In addition, to assess the specific contribution of the ensemble regression strategy, we conducted ablation analyses by independently applying LASSO and SGL, and comparing their performance with the integrated scPASI framework. For each disease-phenotype association, the single-cell dataset was randomly partitioned into ten subsets (seed = 123), and repeated modeling was performed to compute the average C-index (for survival phenotypes) or AUC (for binary phenotypes).

For OS phenotypes, the ensemble scPASI model consistently achieved higher C-index values than either LASSO or SGL alone across all experiments. For binary phenotypes, the ensemble approach outperformed single models in three of five datasets (Table 5). In COAD_tumors and NSCLC_tumors, LASSO achieved an AUC of 1.0, whereas SGL and the ensemble model yielded slightly lower but near-perfect values. This likely reflects the strong separability of these datasets; the perfect AUC under LASSO may indicate partition-specific overfitting, while the ensemble configuration demonstrates more balanced and stable behavior.

**Table 5:** Comparison of single and integrated regression models on C-index (OS) and AUC (binary phenotypes)

Evaluation	Dataset	SGL	LASSO	SGL_LASSO
C-index	BRCA_Surv	0.573553	0.575862	0.5789320
	LIHC_Surv	0.757431	0.761573	0.7644260
	LUAD_Surv	0.693486	0.690243	0.6971020
AUC	COAD_tumors	0.9787003	1	0.9787003
	HNSC_TP53	0.7243738	0.7623237	0.7641734
	LUAD_TP53	0.8082891	0.8517441	0.8524409
	NSCLC_tumors	0.9900518	1	0.9900518
	LUAD_status	0.6331394	0.6158306	0.6360399

The contribution of the Res-VAE module was further assessed by performing ablation experiments in which PFM-derived embeddings were fed directly into the correlation and regression modules, bypassing Res-VAE refinement. Comparative analyses were conducted across eight disease-phenotype datasets, and predictive performance was evaluated using C-index for overall survival (OS) phenotypes and AUC for binary phenotypes (Table 6). The incorporation of the Res-VAE module improved AUC/C-index values in most datasets. Notably, in the NSCLC_tumors dataset, the AUC increased from 0.50 without Res-VAE to 0.9913 with Res-VAE. A slight decrease was observed only for the NSCLC_status dataset, which reflects the patient’s final survival status and may be influenced by multiple clinical and biological factors. In contrast, when evaluating a tumor-specific phenotype within the same disease (NSCLC_tumors), the inclusion of Res-VAE led to a substantial improvement.

**Table 6:** Comparison for binary phenotypes with and without Res-VAE on C-index (OS) and AUC (binary phenotypes)

Evaluation	Dataset	Without Res-VAE	With Res-VAE
C-index	BRCA_Surv	0.5810	0.5838
	LIHC_Surv	0.6967	0.7763
	LUAD_Surv	0.6761	0.6825
AUC	NSCLC_Tumors	0.5000	0.9913
	COAD_Tumors	0.9721	0.9761
	LUAD_TP53	0.7951	0.8109
	HNSC_TP53	0.7125	0.7265
	NSCLC_Status	0.6453	0.6310

In summary, integrating PFM-derived feature embeddings allows scPASI to distinguish SP and WP tumor cell subpopulations, capturing underlying differences in tumor purity and malignancy that reflect the strength of phenotypic associations. This process is supported by the Leiden algorithm, which provides prior cell grouping labels through unsupervised clustering, forming the basis for phenotype-association analysis. The *resolution* parameter, empirically set to a default of 0.5 within an optimal range of 0.4–0.6, controls cluster granularity: lower values yield broader clusters and higher values produce finer partitions. The SGL_LASSO module further enhances scPASI’s performance by delivering lower false positive rates than other regression models, improving robustness and biological relevance. Ablation analyses demonstrated that while LASSO or SGL alone achieved competitive performance, the ensemble integration consistently improved stability across repeated experiments and provided more balanced predictive behavior. Notably, individual regression models can only classify cells into positive or negative associations, whereas their integration enables refined stratification into SP, WP, SN, and WN subpopulations based on both the direction and magnitude of regression coefficients. This fine-grained phenotype classification improves interpretability and strengthens the biological relevance of phenotype-associated cell identification. Together, these components enable scPASI to detect true tumor cells consistently while strengthening the interpretability and reliability of identified phenotype-associated subpopulations.

### scPASI reveals tumor-associated functional traits across cell subpopulations

**Fig. 3:**
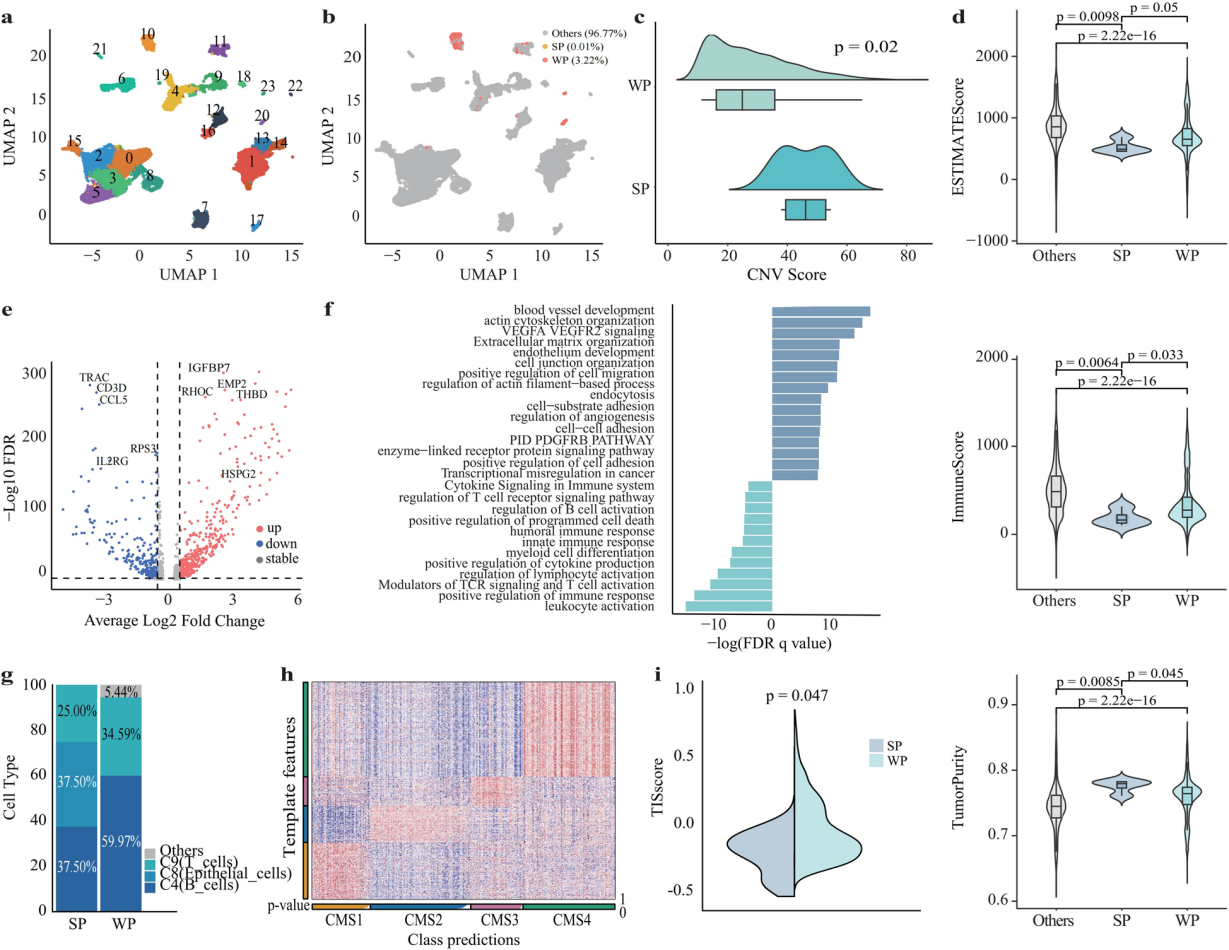
scPASI reveals tumor-associated functional traits in NSCLC and COAD cells. **a** UMAP visualization of 52,698 NSCLC cells colored by Leiden clusters. **b** UMAP plot of scPASI + and scPASI − cells colored by SP (yellow) and WP (red) cells. **c** Comparison of SP and WP cells in terms of copy number variation (CNV) scores. SP cells exhibit significantly elevated CNV levels (*p* = 0.02, two-tailed Wilcoxon rank-sum test). **d** Violin plots comparing ESTIMATE, immune, and stromal scores across SP, WP, and other cells. SP cells show significantly lower ESTIMATE and immune scores and higher tumor purity (all *p* < 0.05, two-tailed Wilcoxon rank-sum test). **e** Volcano plot of differentially expressed genes (DEGs) between scPASI + and other cells. Significantly upregulated (red) and downregulated (blue) genes are indicated (FDR < 0.05, |log2 fold change|> 0.5). **f** Pathway enrichment analysis for DEGs. The bar plot shows significantly enriched biological pathways. **g** Proportional composition of major cell types within SP and WP cells. **h** The heatmap of CMS classification indicates distinct molecular subtype affiliations. **i** Comparison of TIS scores between SP and WP cells. WP cells show significantly higher TIS scores (*p* = 0.047)

Tumor tissues comprise phenotypically diverse malignant subpopulations alongside normal cellular components. Identifying tumor-associated subpopulations is critical for mapping tumor hierarchy and elucidating tumor-microenvironment interactions [38]. Using bulk RNA-seq data from TCGA-LUAD (471 bulk samples) and TCGA-LUSC (504 bulk samples), each annotated with tumor status, scPASI was applied to identify tumor cell subpopulations within 52,698 cells from the NSCLC scRNA-seq dataset. These cells were partitioned into 24 clusters (Fig. 3a), underscoring the heterogeneity of tumor cells. Among the cells identified by scPASI (scPASI + and scPASI − cells), scPASI successfully distinguished a small subset of SP cells alongside a moderate fraction of WP cells, demonstrating its sensitivity in capturing variations in the strength of phenotypic associations. SP cells exhibited a strong positive correlation with tumor status, while WP cells displayed a weak positive correlation (Fig. 3b). To further dissect differences between SP and WP cells, CNV scores were compared, revealing significantly higher CNV levels in SP cells than in WP cells (Fig. 3c). Given that high CNV burden is associated with genomic instability and aggressive tumor behavior, this suggests that SP cells may represent a more malignant state. In addition, SP cells had significantly lower ESTIMATE and immune scores compared to WP cells (Fig. 3d), supporting higher tumor purity and a more malignant phenotype in the SP cell group. Since ESTIMATE was originally developed for bulk RNA-seq data, its direct application at the single-cell level has inherent limitations. To further validate the biological characteristics of the SP and WP subpopulations at single-cell resolution, we additionally examined the expression of tumor malignancy-associated genes (S100A10, EMP2, and RHOC) and immune-related marker genes (B2M, CCL5, and CD3D). S100A10 has been implicated in tumor invasion and metastasis through its role as a plasminogen receptor that mediates plasminogen activation and extracellular matrix degradation, promoting invasive behavior in multiple cancers [39, 40]. EMP2 promotes tumor progression and angiogenesis by enhancing VEGF expression and neo-vascularization in tumor xenografts [41, 42]. RhoC, a member of the Rho GTPase family, regulates cytoskeletal remodeling and cell motility, and its overexpression is associated with enhanced metastatic potential across diverse cancers [43, 44]. In contrast, B2M is a critical component of the MHC class I complex required for antigen presentation to CD8 + T cells [45], CCL5 acts as a chemotactic cytokine recruiting immune effector cells including T cells and monocytes to sites of inflammation or tumors [46], and CD3D forms part of the T-cell receptor (TCR) complex necessary for T-cell activation and signaling [47]. The results show that SP and WP subpopulations exhibit markedly higher expression of tumor malignancy markers (Additional file 6, Figure S2) and lower expression of immune markers compared to background cells (Additional file 7, Figure S3), indicating that the identified subpopulations are highly tumor-specific with low immune activity, consistent with their high-malignancy, low-immune phenotype. These results validate scPASI’s ability to identify cellular heterogeneity differences by capturing variations in the strength of phenotype associations.

Gene expression and functional analyses were compared between scPASI-positive (denoted as scPASI +) cells, which were identified by the scPASI framework as exhibiting the target tumor phenotype and comprising both strongly positive (SP) and weakly positive (WP) subpopulations, and other cells, including non-malignant cells and tumor cells not detected by scPASI. This comparison aimed to elucidate transcriptional patterns specific to scPASI-recognized tumor cells. As a result, 439 upregulated genes and 216 downregulated genes were differentially expressed in scPASI + cells over all other cells, respectively (Fig. 3e). Among these, IGFBP7, which is previously reported to be upregulated in NSCLC, is associated with tumor metastasis and increased lymphatic vascular density, suggesting a potential role in promoting lymphangiogenesis and tumor progression [48]. TRAC and CD3D, which encode essential components of the T cell receptor complex and play key roles in T cell-mediated anti-tumor immunity [49], were markedly downregulated in scPASI + cells. This observation implies impaired T cell infiltration or function, potentially facilitating tumor immune evasion. Functional enrichment analysis further revealed activation of pathways related to vascular development, including VEGFA-VEGFR2 signaling and blood vessel development [50], whereas immune-related pathways such as positive regulation of immune response and T cell receptor signaling were suppressed in scPASI + cells (Fig. 3f).

Furthermore, leveraging the comprehensive molecular subtype annotations available in the COAD dataset, scPASI was applied to identify strongly positive (SP) and weakly positive (WP) cell subpopulations, enabling a detailed comparison of their biological features. COAD samples are categorized into four consensus molecular subtypes (CMS1-CMS4): CMS1 is characterized by high microsatellite instability and pronounced immune infiltration; CMS2 exhibits canonical epithelial features with activation of WNT and MYC signaling; CMS3 shows prominent metabolic reprogramming; and CMS4 features stromal activation and an epithelial-mesenchymal transition (EMT) phenotype [51]. Analysis of cellular composition revealed that SP cells were predominantly epithelial in origin, whereas WP cells consisted largely of immune cells (Fig. 3g). Subtype prediction performed on COAD bulk RNA-seq samples using *CMScaller* [52] indicated a predominance of the CMS2-CMS4 subtypes (Fig. 3h). SP cells exhibited a significantly lower Tumor Inflammation Signature (TIS) score than WP cells (*p* = 0.047, Fig. 3i), suggesting diminished inflammation activity and immune engagement within SP cells.

These findings demonstrate that scPASI identifies distinct cell subpopulations, characterized by strong (SP) and weak (WP) associations to tumor phenotypes, which exhibit pronounced biological differences. These differences are reflected in elevated CNV scores and altered tumor immune microenvironment (TIS) scores among the subpopulations. Furthermore, differential gene expression and pathway enrichment analyses reveal functional distinctions related to tumor malignancy and immune regulation, underscoring the biological relevance of the phenotype-associated subpopulations identified by scPASI.

### scPASI identifies the TP53 mutation-driven divergent molecular mechanisms across cell subpopulations

**Fig. 4:**
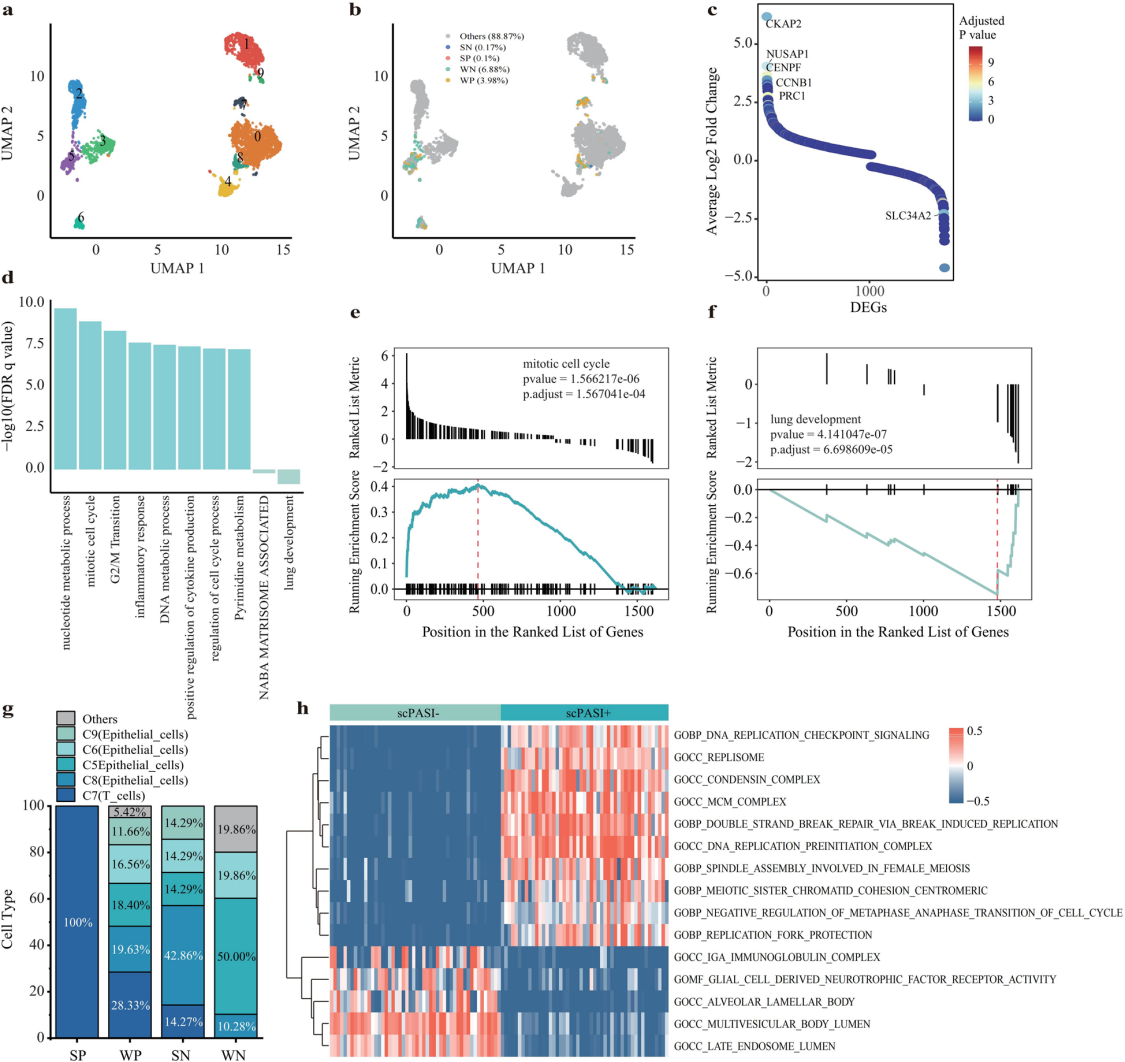
scPASI identifies TP53 mutation-driven molecular mechanisms in LUAD cells. **a** UMAP visualization of 4,102 LUAD cells colored by Leiden clusters. **b** UMAP plot of scPASI + and scPASI − cells colored by SN (blue), SP (red), WN (green), and WP (yellow) cells. **c** Differentially expressed genes (DEGs) between scPASI + and scPASI − cells. The volcano plot highlights the top DEGs ranked by average log2 fold change and adjusted *p*-value. **d** Bar plot of the enriched pathways from DEGs. **e** The gene set enrichment analysis (GSEA) plot shows upregulation of mitotic cell cycle-related gene sets in scPASI + versus scPASI − cells. **f** The GSEA plot shows downregulation of the lung development pathway in scPASI + versus scPASI − cells. **g** Cell type composition of SP, WP, SN, and WN cells. The plot highlights differences in cellular heterogeneity. **h** Heatmap of pathway activity scores calculated by gene set variation analysis (GSVA). Differential pathway activities were identified using the *limma* package (FDR < 0.05), highlighting significantly enriched or suppressed biological processes between scPASI + and scPASI − cells

TP53, one of the most frequently mutated tumor suppressor genes in human cancers [53], plays a central role in maintaining genomic integrity. Identifying cell subpopulations with TP53 mutations in LUAD reveals key malignant clones marked by genomic instability and immune evasion, offering critical insights into tumor heterogeneity and microenvironment dynamics that underlie disease progression and therapeutic resistance [54, 55]. scPASI was further applied to evaluate its capability in identifying cell subpopulations (scPASI + and scPASI −) directly associated with TP53 mutation status and to explore the extent to which these subpopulations reflect molecular mechanisms linked to TP53 dysfunction. First, the LUAD scRNA-seq dataset was integrated with TP53 mutation-annotated bulk RNA-seq data from TCGA-LUAD to facilitate the identification of cell subpopulations among 4102 lung cancer cells, which were grouped into 10 distinct clusters by the Leiden algorithm. Then these clusters can be used as prior information to guide the computation of regression coefficients in the SGL model (Fig. 4a). Finally, by integrating the computational results described above with TP53 mutation information from TCGA-LUAD bulk data, scPASI successfully identified both scPASI + and scPASI − subpopulations among 4102 lung cancer cells. Results show that scPASI identified 167 scPASI-positive (denoted as scPASI +) cells linked to the TP53 mutant phenotype (mainly consisting of WP cells and a small fraction of SP cells), and 289 scPASI-negative (denoted as scPASI −) cells connected to the wild-type (predominantly WN cells, with a few SN cells) (Fig. 4b).

To investigate the transcriptional biology differences between scPASI + and scPASI − cells, gene expression was compared among these cells, revealing 36 upregulated and 24 downregulated genes in scPASI + cells compared with scPASI − cells (Fig. 4c). The upregulated differentially expressed genes in scPASI + cells compared with scPASI − cells were cell cycle- and proliferation-related genes. Among these genes, CKAP2, CENPF, and CCNB1 are key regulators of mitotic progression and cell division [56]. Additionally, the upregulation of these three genes indicates enhanced proliferative capacity and dysregulated cell cycle progression, hallmarks of malignant transformation. Conversely, downregulated genes such as SLC34A2 suggest a loss of alveolar type II cell differentiation markers, supporting a shift toward a dedifferentiated, highly proliferative phenotype and potential remodeling of the local lung microenvironment [57] (Fig. 4c).

Functional enrichment analysis in these DEGs shows the activation of cell cycle-related pathways, such as mitotic cell cycle and regulation of cell cycle process, alongside the suppression of pathways associated with alveolar function, including lung development (Fig. 4d). Gene Set Enrichment Analysis (GSEA) of all differentially expressed genes further revealed significant enrichment of the mitotic cell cycle pathway (Fig. 4e). We also observed a marked suppression of genes involved in lung development (Fig. 4f). Analysis of cellular composition showed that SP cells consisted predominantly of T cells, while WP and SN cells contained a mix of T cells and epithelial cells. In contrast, WN cells were composed primarily of epithelial cells (Fig. 4g). Gene set variation analysis (GSVA) was performed to assess pathway activity quantitatively by comparing scPASI + and scPASI − cell groups using the *limma* package for differential activity testing. Pronounced differences were observed: scPASI + cells exhibited elevated activity in core mechanisms governing cell cycle regulation, DNA replication and repair, and chromosome segregation, whereas scPASI − cells showed higher activity in pathways related to alveolar epithelial secretory functions (Fig. 4h).

These results demonstrate that integrating TP53 mutation information from bulk data into scPASI strengthens the association between identified cell subpopulations and TP53 mutational status. Differential gene expression analysis further elucidated molecular mechanisms underlying TP53 mutation, thereby providing insights into TP53-related tumor evolution and microenvironmental reprogramming.

### High-risk signature derived from scPASI-identified subpopulations predicts adverse prognosis

**Fig. 5:**
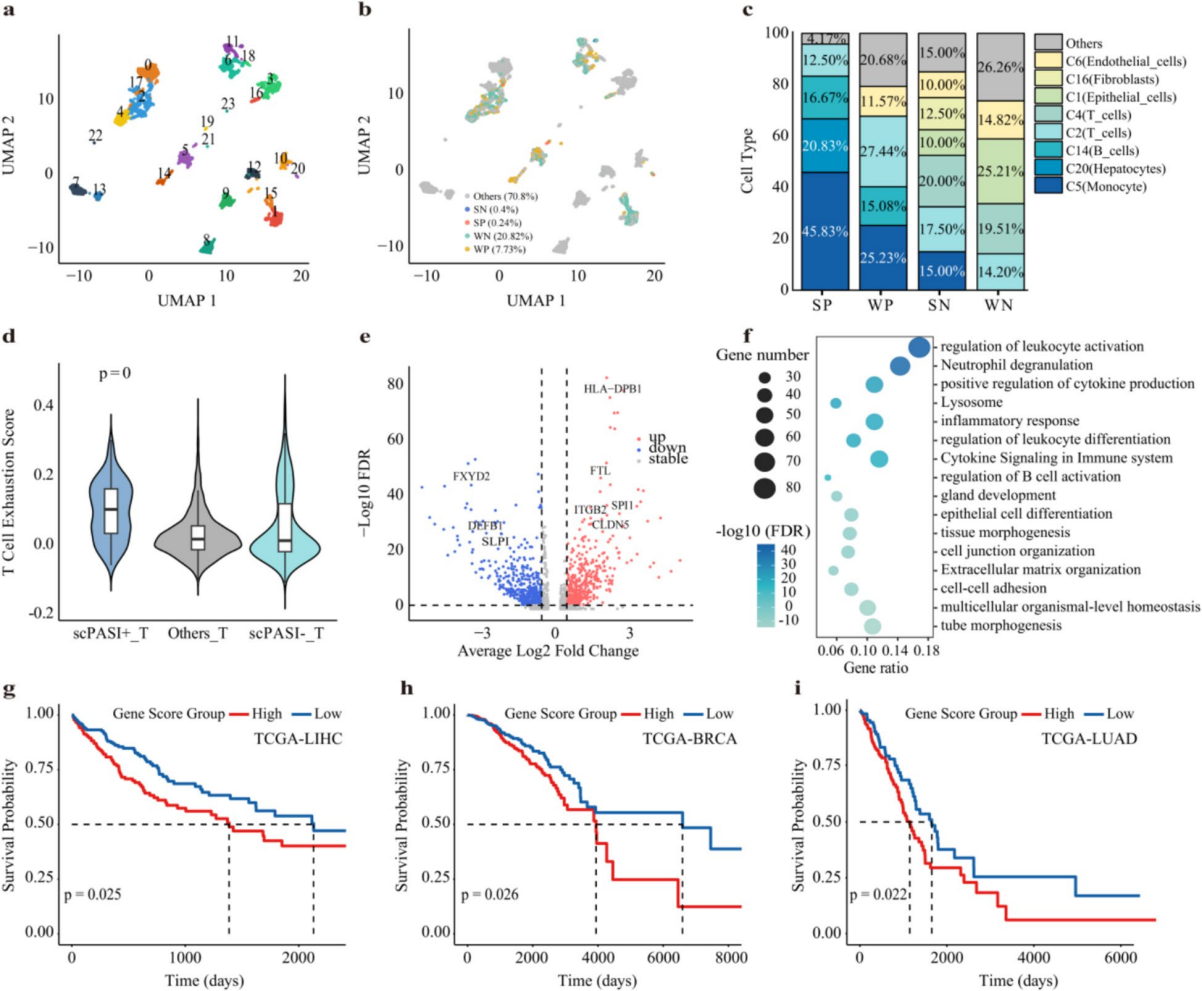
scPASI-derived high-risk signature predicts adverse prognosis in LIHC, BRCA, and LUAD cells. **a** UMAP visualization of LIHC cells. **b** UMAP plot of scPASI + and scPASI − LIHC cells, colored by SN (blue), SP (red), WN (green), and WP (yellow) cells. **c** The proportion of major cell types within SP, WP, SN, and WN groups illustrates differences in cellular heterogeneity. **d** Comparisons of T cell exhaustion scores among scPASI + _T, scPASI − _T, and other cells. The violin plot shows significantly elevated exhaustion in scPASI + _T cells (*p* = 0). **e** Volcano plot of differentially expressed genes (DEGs) between scPASI + and scPASI − cells with significantly upregulated (red) and downregulated (blue) genes highlighted. **f** Bubble plot of enriched pathways from DEG analysis with point size indicating the number of genes and color representing the statistical significance. **g**–**i** Kaplan–Meier (KM) survival analyses for **g** TCGA-LIHC, **h** TCGA-BRCA, and **i** TCGA-LUAD cohorts stratified by high and low gene score groups. Log-rank test *p*-values indicate significant separation between survival curves

Identifying cell subpopulations associated with poor prognosis at the single-cell level offers valuable insights into high-risk components within tumor ecosystems. It clarifies tumor-microenvironment interactions that drive malignant progression [58]. To further evaluate the performance of scPASI in overall survival (OS) data, it was applied to scRNA-seq datasets from LIHC, BRCA, and LUAD to identify cell subpopulations associated with adverse clinical outcomes. Using OS data from 367 TCGA-LIHC bulk samples, scPASI was applied to identify aggressive cancer cell subpopulations within 9946 cells from a corresponding LIHC scRNA-seq dataset. The cells were grouped into 24 clusters (Fig. 5a), highlighting cellular heterogeneity. Of 793 scPASI + cells, a small subset showed a strong positive correlation with poor prognosis (SP cells), while a larger fraction exhibited a weak positive correlation (WP cells). Among 2111 scPASI − cells, a minority were strongly associated with favorable survival (SN cells), and most showed a weak correlation with better prognosis (WN cells) (Fig. 5b). These subpopulations consisted primarily of monocytes, hepatocytes, B cells, T cells, and epithelial cells (Fig. 5c). Given the abundance of T cells, T cell exhaustion scores were calculated, revealing significantly higher exhaustion in scPASI + and scPASI − T cells (Fig. 5d).

To elucidate the underlying transcriptional patterns of scPASI + cells, their gene expression profiles were compared to those of scPASI − cells. This analysis identified 477 upregulated and 469 downregulated genes differentially expressed in scPASI + cells compared with scPASI − cells (Fig. 5e). Among these, SPP1 expression correlated strongly with the infiltration of multiple immune cell types, while KRT19 appeared to modulate immune responses through regulation of cellular architecture and inflammatory signaling [59, 60]. Collectively, these genes contribute to the establishment of an immunosuppressive tumor microenvironment. Functional enrichment analysis further revealed significant activation of immune-related pathways in scPASI + cells, including regulation of leukocyte activation, cytokine signaling, and inflammatory response [61] (Fig. 5f).

To further examine the clinical relevance of the above 477 overexpressed genes (defined as the liver hepatocellular carcinoma signature), these genes were ranked by expression level, and the top 15 genes were used to stratify samples into high- and low-risk groups. In the TCGA-LIHC dataset, patients with higher signature scores showed significantly worse survival time than those with lower signature scores (Fig. 5g). Cox proportional hazards models were also applied to the BRCA and LUAD datasets following the same workflow. Consistent with the LIHC results, higher expression of the top 15 signature genes in each cancer type was significantly associated with shorter overall survival (Fig. 5h, i).

These results demonstrate that a high-risk transcriptional signature originating from genes overexpressed in scPASI + cell subpopulations effectively stratifies patients into distinct prognostic groups across multiple cancer types. Its consistent association with shorter overall survival in LIHC, BRCA, and LUAD cohorts underscores the biological relevance and clinical utility of scPASI-identified cell subpopulations in prognostic stratification.

### scPASI identifies cellular signatures related to clinical outcomes

**Fig. 6:**
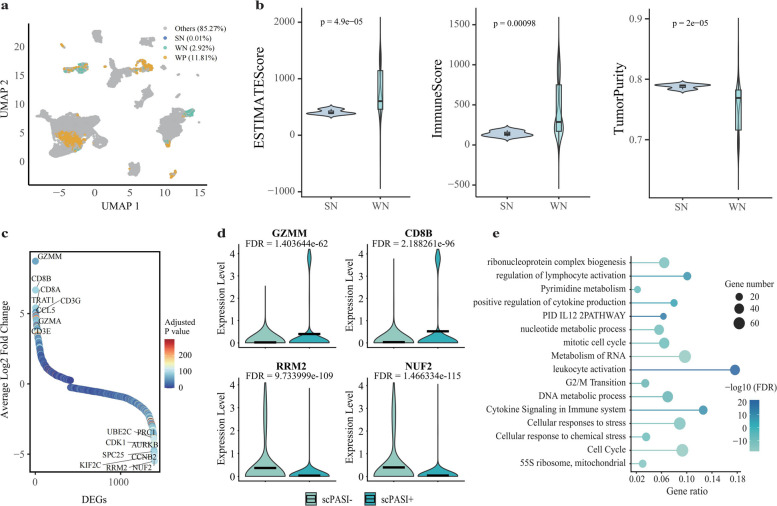
scPASI identifies cellular signatures related to clinical outcomes in NSCLC cells. **a** UMAP visualization of scPASI + and scPASI − cells. **b** Violin plots comparing ESTIMATE, immune, and stromal scores between SN and WN cells. SN cells show significantly lower ESTIMATE and immune scores and higher tumor purity (all *p* < 0.05, two-tailed Wilcoxon rank-sum test). **c** Differentially expressed genes (DEGs) between scPASI + and scPASI − cells. **d** Violin plots of expression levels of selected upregulated and downregulated genes in scPASI + cells. The adjusted *p*-values (FDR) were calculated using the two-tailed Wilcoxon rank sum test. **e** Bubble plot of enriched pathways from DEG analysis with point size indicating the number of genes and color representing the statistical significance

Identifying cell subpopulations correlated with clinical outcomes (i.e., alive or deceased) facilitates the characterization of cellular heterogeneity and offers insights into the diverse cellular compositions that may influence disease progression [62]. This analysis can reveal tumor-promoting or protective cell types, thereby clarifying mechanisms underlying patient survival and highlighting potential targets for therapeutic intervention. The survival endpoint was defined as a binary outcome based on vital status at the last follow-up: deceased patients were classified as having experienced the event. At the same time, those still alive were considered event-free survivors. Using alive (1) versus deceased (0) as the binary phenotype, scPASI identified 6,223 scPASI + cells associated with being alive (all classified as WP) and 1540 scPASI − cells associated with death, which consist primarily of WN cells with few SN cells (Fig. 6a). Within scPASI − cells, SN cells exhibited significantly lower ESTIMATE and immune scores than WN cells (Fig. 6b), suggesting higher tumor purity and potentially a greater influence on patient mortality.

Their gene expression profiles were compared to investigate transcriptional differences between scPASI + and scPASI − cells, revealing 257 upregulated and 740 downregulated differentially expressed genes (Fig. 6c). The top upregulated differentially expressed genes in scPASI + cells were predominantly T cell-associated genes, such as GZMM, CD8B, CD3G, CD8A, and CCL5, which enhance T-cell signaling molecule expression and indicate heightened immune activity [63]. In contrast, the downregulated genes were mainly involved in mitosis, DNA synthesis, and repair, including RRM2, NUF2, AURKB, and UBE2C, reflecting enhanced proliferative capacity characteristic of tumor cells [64, 65] (Fig. 6c). Some upregulated genes encoded critical T cell-associated markers and cell proliferation-related regulators (Fig. 6d). Pathway enrichment analysis confirmed strong activation of immune-related pathways in scPASI + cells, such as regulation of lymphocyte activation and T cell-mediated immunity, indicating enhanced T-cell activation [66]. Conversely, pathways such as G2/M Transition and DNA metabolic process were suppressed, indicating reduced activity in DNA replication, mitosis, and nucleic acid synthesis. These processes represent proliferative signals commonly enriched in highly proliferative tumor cells [67, 68] (Fig. 6e).

In summary, by leveraging clinical outcomes, scPASI successfully identifies and characterizes distinct cell subpopulations associated with divergent clinical prognoses. SN cells showed elevated tumor purity, suggesting a more substantial influence on mortality, potentially mediated through microenvironment modulation. Transcriptional profiling further uncovered an enrichment of T cell-related genes and suppressive proliferative pathways within alive-status-associated subpopulations. These findings underscore the capacity of scPASI to elucidate clinically meaningful cellular heterogeneity and reveal mechanistic signatures that underlie patient survival outcomes.

## Discussion

In this study, the key innovations of scPASI can be summarized as follows: (1) Feature extraction using pre-trained foundation models, which provides biologically informed and transferable embeddings to enhance robustness and cross-dataset generalizability; (2) Refinement with Res-VAE, stabilizing cell-sample similarity and preserving phenotype-relevant structural variation for reliable downstream modeling; (3) Integration of LASSO and SGL regression, enabling interpretable and structured identification of phenotype-associated cell subpopulations; and (4) Fine-grained stratification of associations, classifying cells into strong/weak positive and strong/weak negative categories for nuanced characterization of cellular heterogeneity.

scPASI exhibited comparable sensitivity and stability in capturing subtle phenotype associations compared to alternative regression combinations and existing approaches. This advantage was driven by integrating scFoundation, Res-VAE, and regression models, each contributing to enhanced robustness and transferability. Notably, the refined stratification into strong and weak associations offered a more nuanced characterization of tumor heterogeneity than conventional dichotomous frameworks. As with any coefficient-based categorization, potential uncertainty may arise near threshold boundaries, particularly for cells with regression coefficients close to zero or predefined cutoffs distinguishing strong from weak associations. In such cases, the distinction between adjacent categories may be less stable. Therefore, in practical applications, we recommend interpreting boundary cases cautiously and considering the magnitude of regression coefficients, statistical significance, and biological context jointly when evaluating phenotype relevance. Moreover, by incorporating bulk RNA-seq data, scPASI consistently identified phenotype-associated subpopulations across diverse single-cell disease datasets, underscoring both the detected subgroups' biological precision and clinical relevance.

Although scPASI performs strongly in identifying phenotype-associated cell subpopulations and exploring the key transcriptional signatures from single-cell genomics, the current implementation primarily focuses on linking single-cell transcriptomic features with bulk-level phenotypes. This may restrict its ability to capture the full spectrum of cell–phenotype associations due to the lack of multi-omics information. This limitation could result in an underestimation of subtle yet biologically relevant signals.

Moreover, extending scPASI to incorporate additional omics layers, such as scATAC-seq or spatial transcriptomics, would enhance data diversity and broaden its applicability across various biomedical contexts. Approaches like scMultiomeGRN [69] demonstrate the feasibility and value of integrating chromatin accessibility data with transcriptomic profiles. Another promising direction is to extend scPASI by incorporating genome-wide association study (GWAS) [70] data. GWAS summary statistics can be projected to the gene level and subsequently used as phenotype inputs for scPASI, enabling the identification of cell subpopulations that are enriched for genetic risk signals. In addition, GWAS data can serve as an external validation resource by testing whether subpopulation-specific signatures show significant enrichment of disease-associated loci.

While scPASI demonstrates strong performance across diverse datasets, its advantages are most evident in scenarios characterized by complex phenotype structures, cross-cohort heterogeneity, or subtle cell-phenotype associations that may not be captured by clustering-based or purely unsupervised approaches. In particular, when linking single-cell transcriptomic heterogeneity to multifaceted phenotypes, the integration of PFM-based feature extraction, dimensionality reduction via Res-VAE to obtain transferred embeddings, and phenotype-guided regression with structured sparsity provides enhanced robustness and interpretability. However, in relatively simple settings where cell populations are clearly separable and phenotype labels correspond to well-defined transcriptional differences, conventional clustering-based methods or simpler regression frameworks may be sufficient and computationally more efficient. In addition, the framework depends on the quality and transferability of PFMs, which may influence performance in highly domain-specific datasets. A minor limitation of the current scPASI implementation is the mixed use of Python and R, which may introduce slight inconsistencies in data processing and increase computational workflow complexity; future work will focus on unifying the framework under a single programming language to streamline workflows and improve reproducibility.

## Conclusions

In summary, scPASI offers a robust framework for integrating scRNA-seq data with bulk RNA-seq data, enabling (1) accurate identification of phenotype-associated cell subpopulations by combining diverse phenotype data such as tumor status, gene mutations, survival outcomes, (2) identification of key genes and enriched pathways among scPASI-identified cell populations, and (3) prognostic and functional evaluation, including survival analysis, tumor purity, and tumor malignancy, to guide prognosis and targeted therapies. By enhancing the biological relevance, scPASI holds strong potential to accelerate disease-related discoveries. It is anticipated to facilitate the broad utilization of widely available phenotype data in single-cell analysis and to uncover the most disease-relevant subpopulations, ultimately informing cell-targeted therapeutic strategies.

## Methods

### Datasets

To facilitate integrative analysis of bulk and single-cell transcriptomic datasets, we curated data across six cancer types, including Non-Small Cell Lung Cancer (NSCLC), Lung Adenocarcinoma (LUAD) [71], Colon Adenocarcinoma (COAD) [72], Liver Hepatocellular Carcinoma (LIHC) [73], Breast Invasive Carcinoma (BRCA) [74], and Head and Neck Squamous Cell Carcinoma (HNSC) [75]. Bulk RNA-seq data together with corresponding clinical information were retrieved from The Cancer Genome Atlas (TCGA) via the Genomic Data Commons portal. For the NSCLC dataset, bulk RNA-seq profiles were constructed by combining samples from the Lung Squamous Cell Carcinoma (LUSC) [76] and LUAD cohorts.

For the single-cell analysis of NSCLC aimed at identifying tumor-cell subpopulations and those associated with binary survival outcomes (alive versus deceased), scRNA-seq data were acquired from ArrayExpress (accession numbers: E-MTAB-6149 and E-MTAB-6653) [77]. The merged dataset comprised 52,698 cells, including 13,375 tumor cells and 39,323 normal cells. Additionally, to investigate subpopulations linked to poor prognosis and TP53 mutation status, a subset of 4102 tumor cells was extracted from the LUAD dataset.

For the COAD analysis, scRNA-seq data were downloaded from the Gene Expression Omnibus (GEO) under accession code GSE132465 [78]. This dataset contained 63,689 cells from 23 patient samples, including 47,285 tumor cells and 16,404 normal cells. To reduce computational burden while maintaining representativeness, we performed random downsampling (with a fixed seed of 123) and retained 10% of tumor and normal cells for downstream analysis.

In the LIHC analysis, scRNA-seq data used for survival-associated subpopulation identification were obtained from GEO (accession code: GSE125449) [79]. Similarly, for the BRCA survival analysis, the scRNA-seq data from GEO (accession code: GSE161529) were utilized [80]. These datasets were processed consistently using the scPASI framework to identify clinically relevant subpopulations. For performance comparison between different regression model combinations and existing methods based on AUC values, HNSC scRNA-seq data were obtained from GEO (accession code: GSE103322) [81].

### Overall framework of scPASI

The scPASI (single-cell Phenotype-associated Subpopulation Identification) framework includes four sub-modules: PFM-based feature extraction module, Res-VAE transfer learning module, statistical feature learning module, and cell phenotype identification module. It integrates data from three sources: a single-cell RNA sequencing (scRNA-seq) dataset containing $m$ cells, a bulk RNA-seq dataset from $n$ samples, and a phenotype variable $Y$ representing disease-related clinical information (e.g., binary classification or overall survival). The scRNA-seq and bulk data are processed through the PFM module to extract feature embeddings, while the sample phenotype $Y$ annotates each bulk sample. These phenotype annotations are subsequently utilized in the cell phenotype identification module alongside the transferred cell embeddings and the transferred sample embeddings derived from previous modules, forming the basis for regression modeling. Specifically, the cell phenotype identification module incorporates both LASSO and SGL regression models, in which the transferred embeddings and cell clusters defined by the Leiden algorithm are used to calculate regression coefficients. By evaluating the sign of these coefficients, four phenotype-associated subpopulations are identified: strongly positive (SP), weakly positive (WP), strongly negative (SN), and weakly negative (WN) cells. Finally, the identified cell subpopulations are subjected to cell phenotype-associated downstream analysis. The framework of scPASI is presented in Fig. 1.

### PFM-based feature extraction module

In this study, scFoundation [23], a foundation model developed for transcriptomic data and pre-trained on over 5 million single-cell expression profiles, is utilized as the feature representation model for extracting cell and sample feature embeddings. Built upon the xTrimoGene [82] architecture with 100 million parameters, it captures intrinsic and transferable gene expression patterns across various biological contexts, thereby rendering it particularly suitable for downstream analyses related to cell phenotypes. The resulting feature embedding matrix $X$ should be $X\in {\mathbb{R}}^{N\times F}$, where $N$ represents the number of samples in bulk expression data or the number of cells in scRNA-seq data, and $F$ represents the feature embedding dimension.

Following the scFoundation implementation, feature embeddings are derived using max-mean pooling across all gene expression values, and further concatenated with the embeddings of two special tokens, T and S, representing the total gene expression count. This process yields a fixed feature embedding dimension of 3072. scFoundation supports a maximum vocabulary size of 19,264 genes, ensuring broad coverage across transcriptomic datasets.

### Res-VAE transfer learning module

The Res-VAE model adopts a fully connected encoder-decoder architecture, centered around a variational inference layer [83]. The encoder and decoder use specially designed residual blocks [84] to enhance training stability and feature expressiveness [85]. Each residual block comprises a batch normalization layer and an adaptive dimensionality matching mechanism. The block performs identity mapping when the adjacent layers’ input and output dimensions are identical. When dimensions differ, a fully connected layer without activation is applied to align them.

The variational inference layer follows the standard-VAE design, comprising two parallel fully connected networks: one estimates the mean $u$, and the other estimates the log variance $log\_var$. The transferred representation vector $Z$ is approximated with the following formula:

1 $$\mathrm Z=\mathrm u+\mathrm\varepsilon\bullet\exp(\frac12\bullet\log\_\mathrm{var})$$

where $\varepsilon$ is sampled from a standard normal distribution of $\exp(\frac12\bullet\log\_\mathrm{var})$; this is known as the Reparameterization Trick, a classical VAE construction method.

In this study, the Res-VAE model is initialized with pre-trained weights from the scATD [86] framework, allowing the model to benefit from prior training on large-scale single-cell data and improve downstream performance.

### Statistical feature learning module

The cell and sample embeddings, represented as 3072-dimensional feature vectors derived from the PFM-based feature extraction module, are inputs to the pre-trained Res-VAE model. The encoder transforms these inputs into 471-dimensional transferred cell embeddings and transferred sample embeddings, respectively.

The Leiden algorithm [87] partitions nodes within a graph based on their pairwise similarity, where each resulting cluster can be interpreted as a group of cells sharing similar molecular characteristics or functional properties. Under the assumption that transcriptionally similar cells are likely to exhibit consistent associations with phenotypic outcomes, it is expected that cells forming densely connected subgraphs (i.e., communities) within the network will display more similar regression coefficients during downstream modeling. This means that cells with similar gene expression profiles tend to have similar effects on the phenotype, as reflected by comparable regression weights, since they often share functional roles or participate in related pathways.

To capture this relationship, a cell–cell similarity network $G$ is constructed using Euclidean distance calculated from the transferred cell embeddings. The Leiden algorithm utilizes an iterative approach to enhance the initial partition by exchanging cells between communities to maximize the modularity score $Q$. This process continues until no further improvement is achievable, ultimately yielding a Leiden cluster label for each cell:

2 $$Q=\frac{1}{2e}\sum_{i,j}[\left({A}_{ij}-\gamma \frac{{k}_{i}{k}_{j}}{2e}\right)\delta ]$$

where $e$ stands for the total number of edges in the graph, ${A}_{ij}$ represents the edge weight between cell $i$ and cell $j$, and $\gamma$ is a *resolution* parameter. In this study, we set the value of $\gamma$ within the range of 0.4 to 0.7, which was empirically determined as the optimal range based on experimental results (see Results for details). ${k}_{i}$ and ${k}_{j}$ are degrees of cell $i$ and cell $j$, respectively. ${c}_{i}$ and ${c}_{j}$ are communities to which cell $i$ and cell $j$ are assigned, respectively. $\delta =1\left({c}_{i}={c}_{j}\right)$, where $1\left(\cdot \right)$ denotes the indicator function that equals 1 if the condition holds and 0 otherwise.

After extracting transferred embeddings from the scRNA-seq and bulk expression data using the PFM and Res-VAE modules, we obtained the transferred cell embeddings and the transferred sample embeddings. Based on these representations, we computed a Pearson correlation matrix $S=({s}_{ij}{)}_{n\times m}$ between each single cell and each bulk sample to quantify the similarity between the transferred cell embeddings and the transferred sample embeddings [88]. Each correlation coefficient ${s}_{ij}$ reflects the similarity between a sample $i$ and a cell $j$ across their shared dimensions in the transferred embedding space.

### Cell phenotype identification module

Combining multiple regression models, the cell phenotype identification module assesses associations between individual cells and specific phenotypes. Each model generates a coefficient vector whose dimension corresponds to the number of cells. Based on the coefficient value assigned to each cell, scPASI determines the direction (positive or negative) and strength (strong or weak) of its association with a given phenotype. This enables the classification of cells into distinct phenotype-associated subpopulations.

This study incorporates an ensemble learning strategy based on the Least Absolute Shrinkage and Selection Operator (LASSO) [31] model and the Sparse Group LASSO (SGL) [32] model into the cell phenotype identification module. The LASSO model introduces an L1 regularization term, which enables effective feature selection in high-dimensional data. The SGL model extends this capability by incorporating both L1 and group L2 regularization terms, allowing for simultaneous identification of sparse variables and group structures.

In this module, three types of data are processed using the LASSO and SGL models: the correlation matrix $S={\left(s_{ij}\right)}_{n\times m}$ constructed from the transferred cell embeddings and transferred sample embeddings, the Leiden cluster assignments, and the sample phenotype *Y*.

The objective functions for LASSO and SGL are defined as follows. The loss function for the LASSO model is:

3 $${\mathrm\beta}_1=\mathrm{argmin}-\frac{1}{\mathrm{n}}l\left(\beta \right)+\mathrm\lambda\parallel\mathrm\beta\parallel_1$$

while the loss function for the SGL model is:

4 $${R}_{G}\left(\beta \right)=\sum_{l=1}^{g}\sqrt{{p}_{l}}\parallel {\beta }^{\left(l\right)}{\parallel }_{2}$$

5 $${\beta }_{2}=\mathrm{arg min}-\frac{1}{\mathrm{n}}l\left(\beta \right)+\lambda \{\alpha \parallel \beta {\parallel }_{1}+\left(1-\alpha \right){R}_{\mathrm{G}}\left(\beta \right)\}$$

where $g$ denotes the number of Leiden clusters, ${p}_{l}$ represents the number of cells in the $l$-th cluster.

In the scPASI framework, the LASSO model is fitted based on the sample phenotype $Y$ and the correlation matrix $S$. In contrast, the SGL model further incorporates the *Leiden* clusters into the optimization process. The vector *β* denotes the regression coefficients associated with individual cells. The formula of the log-likelihood function *l*(*β*) depends on the type of phenotype $Y$. In the scPASI framework, the LASSO regression model is fitted to predict the sample-level phenotype using the correlation matrix between bulk samples and single cells as predictors.

Let ${S}_{i}={\left({s}_{i1},{s}_{i2},...,{s}_{im}\right)}^{T}$ denote the vector of correlation coefficients between sample $i$ and each of the $m$ cells, where ${s}_{ij}$ is the correlation between sample $i$ and cell $j$. If $Y$ is a binary indicator vector, such as ${y}_{i}\in \{\mathrm{0,1}\}$, the logistic regression log-likelihood function is employed in the LASSO model to associate $Y$ with $S$.

6 $$l\left(\beta \right)=\sum_{i=1}^{n}\left[{y}_{i}{\beta }^{T}{S}_{i}-\mathrm{log}\left(1+\mathrm{exp}\left({\beta }^{T}{S}_{i}\right)\right.\right]$$

Furthermore, to incorporate prior cell grouping information (Leiden clusters), the logistic regression log-likelihood function is adopted in the SGL model, which imposes both group-level and individual-level sparsity constraints.

7 $$l\left(\beta \right)=\sum_{i=1}^{n}\sum_{l=1}^{g}\left\{{y}_{i}{\beta }^{\left(l\right)T}{S}_{i}^{\left(l\right)}-\mathrm{log}\left[1+\mathrm{exp}\left({\beta }^{\left(l\right)T}{S}_{i}\right)\right]\right\}$$

For time-to-event outcomes subject to independent censoring, the Cox regression is considered. Let ${T}_{i}$ be the non-negative event time and ${C}_{i}$ be the censoring time. Denote ${\widehat{T}}_{i}=\mathrm{min}\left({T}_{i},{C}_{i}\right)$ as the observed event time or censoring time, and ${\delta }_{i}=I\left({T}_{i}\le {C}_{i}\right)$ as the event indicator, where $I\left(\cdot \right)$ is an indicator function. The following log-likelihood function is used in the LASSO model:

8 $$l\left(\beta \right)=\sum_{i=1}^{n}{\delta }_{i}\left[{\beta }^{T}{S}_{i}-\mathrm{log}\left(\sum_{k\in {R}_{i}}\mathrm{exp}\left({\beta }^{T}{S}_{k}\right)\right)\right]$$

The following log-likelihood function is used in the SGL model:

9 $$l{\left(\beta \right)}_{i}^{\left(l\right)}={\delta }_{i}\{{\beta }^{\left(l\right)T}{S}_{i}^{\left(l\right)}-\mathrm{log}\left[\sum_{k\in {R}_{i}}\mathrm{exp}\left({\beta }^{\left(l\right)T}{S}_{k}^{\left(l\right)}\right)\right]\}$$

10 $$l\left(\beta \right)=\sum_{i=1}^{n}\sum_{l=1}^{g}l{\left(\beta \right)}_{i}^{\left(l\right)}$$

where ${R}_{i}=\left\{k:{\widehat{T}}_{k}\ge {\widehat{T}}_{i}\right\}$ denotes the risk set at time ${\widehat{T}}_{i}$.

LASSO and SGL were independently fitted to the complete dataset under two separate regularization penalties. While LASSO enforces sparsity at the individual feature scale, SGL incorporates structured group constraints, resulting in complementary selection behaviors. To mitigate model-specific bias, a cross-model consensus criterion was applied. Rather than classifying cells solely based on the coefficient sign from a single model, a cell was defined as phenotype-associated only if it received non-zero coefficients in both models with consistent effect directions. This agreement-based filtering enhances the robustness of phenotype-associated cell identification by reducing instability arising from distinct regularization assumptions.

The non-zero coefficients of ${\beta }_{1}$ and ${\beta }_{2}$ solved by the above optimization models are used to select the cell subpopulations associated with the phenotype of interest. A consensus-based selection ensemble strategy is employed to integrate the results from the two independently optimized models. Specifically, the estimated values of ${\beta }_{1}$ (from the LASSO model) and ${\beta }_{2}$ (from the SGL model) are aggregated accordingly. This approach enhances the identification of target cell subpopulations. The specific criteria for determining the phenotype-associated cells are as follows:

11 $$scPAS{I}_{i}=\left\{\begin{array}{cc}SP,{\beta }_{1}>0\hspace{0.25em}\mathrm{and}\hspace{0.25em}{\beta }_{2}>0& \\ WP,{\beta }_{1}>0\hspace{0.25em}\mathrm{or}\hspace{0.25em}{\beta }_{2}>0& \\ SN,{\beta }_{1}<0\hspace{0.25em}\mathrm{and}\hspace{0.25em}{\beta }_{2}<0& \\ WN,{\beta }_{1}<0\hspace{0.25em}\mathrm{or}\hspace{0.25em}{\beta }_{2}<0& \end{array}\right.$$

Here, $scPAS{I}_{i}=SP$ indicates that cell $i$ is strongly positively associated with the phenotype, $scPAS{I}_{i}=WP$ indicates a weak positive association, $scPAS{I}_{i}=SN$ indicates a strong negative association, and $scPAS{I}_{i}=WN$ indicates a weak negative association.

### Cell phenotype-associated downstream analysis

In the Phenotype-associated subtype prediction, we performed single-cell differential expression analysis (scDEA) on scRNA-seq data using the *FindMarkers* function provided by the *Seurat* package (version: 5.3.0) [89], specifying the MAST method [90] to fit a generalized linear model. This approach accounts for common characteristics of scRNA-seq data, such as zero inflation and overdispersion, and facilitates the identification of differentially expressed genes (DEGs) between subpopulations. DEGs were selected based on an absolute log fold change (logFC) greater than 0.5 and a *P*-value less than 0.05. Subsequently, gene set enrichment analysis (GSEA) [91] was conducted to investigate the enrichment of DEGs under various biological conditions. GSEA was implemented using the *gseGO* and *gseKEGG* functions from the *clusterProfiler* package (version 4.14.4) [92]. *P*-values were calculated based on the hypergeometric distribution, and false discovery rates (FDRs) were adjusted using the Benjamini–Hochberg procedure. Survival analysis was performed using the *survival* package (version 3.8.3) and the *survminer* package (version 0.5.0). The top 15 up-regulated genes ranked by logFC were used to stratify samples into high-risk and low-risk groups, and Kaplan–Meier survival curves were plotted to assess prognostic difference.

In the tumor cell identification analysis, cells identified by scPASI as positively associated with tumor phenotypes were defined as tumor cells. Tumor-related properties were then characterized by comparing strongly positive (SP) and weakly positive (WP) cells in terms of tumor purity using the *estimate* package (version 1.0.13) [93]. ESTIMATE (Estimation of STromal and Immune cells in MAlignant Tumor tissues using Expression data) primarily evaluates the abundance of non-tumor components, including immune and stromal cells, in tumor samples. The method applies single-sample gene set enrichment analysis (ssGSEA) to the transcriptome of each tumor sample to calculate StromalScore and ImmuneScore, thereby inferring the levels of infiltrating stromal and immune cells. The sum of these scores constitutes the ESTIMATE score, which can be used to infer tumor purity representing the proportion of tumor cells within the tissue microenvironment, thereby revealing distinct tumor microenvironment characteristics between cell subpopulations. Additionally, large-scale chromosomal copy number variations (CNVs) were inferred for cell subpopulations using the *infercnv* package (version 1.22.0) [94]. CNV scores were calculated to evaluate differences in tumor malignancy across subpopulations.

### Performance evaluation and robustness analysis

To evaluate the performance of scPASI in distinguishing phenotype-associated cell subpopulations and to quantitatively compare it with existing methods, we employed multiple complementary metrics, including the Area Under the ROC Curve (AUC) for binary classification and the Concordance Index (C-index) for survival prediction.

For binary classification tasks, such as distinguishing tumor versus non-tumor cells, the AUC quantifies the model’s overall discriminative ability across all possible thresholds. For each sample $i$, the true phenotype label is ${y}_{i}$, and the predicted score is ${\widehat{y}}_{i}$, representing the maximum probability from the ensemble of LASSO and SGL models. Let $t$ denote a classification threshold used to convert predicted scores into binary labels. In ROC analysis, $t$ is varied across all possible values derived from the predicted scores. For a given threshold t, samples with ${\widehat{y}}_{i}< t$ are classified as positive, whereas those with ${\widehat{y}}_{i}< t$ are classified as negative. The classification outcomes are defined as:

12 $$\begin{array}{cc}TP (True Positive) : & {y}_{i} = 1,{\widehat{y}}_{i} \ge t\\ TN (True Negative) : & {y}_{i} = 0,{\widehat{y}}_{i} < t\\ FP (False Positive) : & {y}_{i} = 0,{\widehat{y}}_{i} \ge t\\ FN (False Negative) : & {y}_{i} = 1,{\widehat{y}}_{i} < t\end{array}$$

The ROC curve is constructed by varying $t$ over all possible values, and the AUC is computed as the area under the curve [95]. Here, AUC was computed exclusively from cross-validated out-of-fold (OOF) predicted probabilities. Specifically, in each outer fold of *K*-fold cross-validation, the model (including $\lambda$ selection via internal cross-validation in *cv.glmnet*/*cvSGL*) was trained using the *K-*1 training folds only, and predicted probabilities were generated solely for the held-out fold. The resulting OOF probabilities were aggregated to produce one prediction per sample. ROC curves and AUC values were then calculated from the continuous OOF scores using the R package *pROC* (version 1.18.5) [96]. Specifically, ROC curves were constructed using *pROC::roc*, which computes sensitivity and specificity across all possible decision thresholds, and AUC was obtained using *pROC::auc*, corresponding to the trapezoidal integration of the ROC curve. No decision threshold was tuned or selected during evaluation. Importantly, AUC captures the model’s global discriminative ability and is independent of any single threshold used for subpopulation classification.

For survival prediction tasks, we used the C-index to assess the agreement between predicted risk scores and observed survival times. For all comparable pairs of patients $(i, j)$ where patient $i$ has a shorter observed survival than patient $j$, the prediction is considered concordant if the predicted risk for $i$ is higher than for $j$. The C-index is defined as the proportion of all usable patient pairs that are concordant, ranging from 0.5 (random prediction) to 1.0 (perfect prediction), and is widely used in censored survival data analysis [97]. In our analysis, survival objects were constructed using the *Surv* function from the R package *survival* (version 3.8.3), and predicted risk scores were computed as linear predictors ($\widehat{\eta } = X\beta$) from the fitted SGL and LASSO models. The C-index was calculated using the *survConcordance* or *concordance* functions implemented in the survival package. For ensemble models, combined risk scores were derived either by averaging linear predictors or by averaging regression coefficients prior to computing linear predictors, and the C-index was calculated accordingly from the resulting combined risk scores.

Due to the availability of true tumor cell annotations in the NSCLC and COAD datasets, we specifically evaluated tumor-associated cell subpopulation identification performance on these two datasets using two cell-level metrics: the Number of Correctly Identified Tumor Cells and the False Positive Rate (FPR). The Number of Correctly Identified Tumor Cells counts the number of cells with true tumor phenotype that are classified as tumor-associated by scPASI or existing methods. This metric directly reflects the model’s ability to capture truly tumor cells at a given decision threshold and provides an intuitive measure of practical identification performance. FPR quantifies the proportion of non-tumor cells that are incorrectly classified as tumor-associated cells at the same threshold. In this cell-level evaluation setting, FP (False Positive) and TN (True Negative) are defined based on individual cell annotations rather than sample-level predictions used in AUC computation. The FPR is calculated as:

13 $$\mathrm{FPR}=\frac{\mathrm{FP}}{(\mathrm{FP}+\mathrm{TN})}$$

Here, $FP$ is defined as a non-tumor cell (${y}_{i} = 0$) that is incorrectly classified as tumor-associated (${\widehat{y}}_{i} = 1$), $TN$ is defined as a non-tumor cell (${y}_{i} = 0$) that is correctly classified as non-tumor (${\widehat{y}}_{i} = 0$).

To evaluate the robustness of subpopulation stratification (SP/WP/SN/WN), a subsampling-based stability analysis was conducted using two representative disease–phenotype datasets (LUAD_TP53 and COAD_tumors). Subpopulation labels were first established by applying regression modeling to the full dataset. Iterations were then defined by progressively removing 10% to 90% of the negatively associated cells, identified from the full dataset, in 10% increments, while retaining all positively associated cells to preserve phenotype-associated structure. This process generated increasingly perturbed versions of the data. For each downsampled dataset, the LASSO and SGL models were refitted and subpopulation categories were reassigned accordingly. Classification stability was subsequently evaluated under increasing degrees of perturbation. Transition rates were calculated to quantify how frequently WP cells shifted to SP or negative categories across subsampling conditions. Low transition rates across downsampling levels indicate robust and stable subpopulation stratification.

The effectiveness of incorporating pre-trained foundation models (PFMs) was assessed through a comparative experiment between PFM-based feature extraction and a non-PFM baseline. The non-PFM baseline followed a standard *Seurat* workflow. First, a Seurat object was created using *CreateSeuratObject*, and low-quality cells and genes were filtered according to minimum cell and gene count thresholds. The expression matrix was then normalized using *NormalizeData* to transform raw counts into comparable expression values, following the specified normalization method and scaling factor. Highly variable genes (HVGs) were identified using *FindVariableFeatures* to capture genes exhibiting substantial cell-to-cell variability for downstream analysis. These HVGs were subsequently standardized using *ScaleData* to remove differences in expression scales across genes. Finally, principal component analysis (*RunPCA*) was applied to reduce the high-dimensional gene expression data into low-dimensional feature vectors, which served as input for correlation computation, clustering, and regression analyses.

To assess the impact of the Res-VAE module within the scPASI framework, ablation experiments were conducted in which PFM-derived feature embeddings were directly fed into the correlation computation and regression modules, bypassing the Res-VAE refinement. The workflow remained consistent with standard scPASI. Comparative analyses were performed across eight disease-phenotype datasets, and performance was assessed using AUC for binary phenotypes and C-index for overall survival phenotypes to quantify the impact of Res-VAE on phenotype-associated cell subpopulation identification.

## Supplementary Information

Additional file 1. Multi-Dimensional Comparison of scPASI and Existing Methods.

Additional file 2. Stability Analysis of WP Cells Under Progressive Downsampling (COAD_tumors).

Additional file 3. Comparison of Leiden and Louvain Clustering Algorithms Across Resolutions on the Number of Correctly Identified Tumor Cells and AUC Values.

Additional file 4. Comparison of Four Regression Model Combinations on the Number of Correctly Identified Tumor Cells and AUC Values.

Additional file 5. Comparison of scPASI with and without PFM in terms of CNV scores for cells identified in the COAD dataset.

Additional file 6. Expression of Tumor Malignancy Markers Across Identified Cell Subpopulations. (a) Expression of Gene S100A10. (b) Expression of Gene EMP2. (c) Expression of Gene RhoC.

Additional file 7. Expression of Immune-Related Markers Across Identified Cell Subpopulations. (a) Expression of Gene B2M. (b) Expression of Gene CCL5. (c) Expression of Gene CD3D.

## Data Availability

All data generated or analysed during this study are included in this published article, its supplementary information files and publicly available repositories. The NSCLC scRNA-seq data are available in the ArrayExpress database (E-MTAB-6149 and E-MTAB-6653 [77]; https://www.ebi.ac.uk/biostudies/arrayexpress), from which the scRNA-seq data for LUAD were derived. Other scRNA-seq datasets used in this study are available in the GEO database (https://www.ncbi.nlm.nih.gov/geo/), including those for COAD (GSE132465 [78]), LIHC (GSE125449 [79]), BRCA (GSE161529 [80]), and HNSC (GSE103322 [81]). Bulk RNA-seq datasets and corresponding phenotype information for six cancer types (LUAD [71], COAD [72], LIHC [73], BRCA [74], HNSC [75], LUSC [76]) were retrieved from the TCGA database (https://portal.gdc.cancer.gov/). The code for this study has been archived in Zenodo at 10.5281/zenodo.19489572 and is also available on GitHub at https://github.com/xnpan/scPASI, while the data used in this study has been archived in Zenodo at 10.5281/zenodo.19490113.

## Abbreviations

scPASI: Single-cell Phenotype-associated Subpopulation Identification
scPASI +: Phenotype-associated positive cells (SP + WP)
scPASI −: Phenotype-associated negative cells (SN + WN)
scRNA-seq: Single-cell RNA sequencing
RNA-seq: RNA sequencing
PFM: Pre-trained foundation model
VAE: Variational autoencoder
Res-VAE: Residual variational autoencoder
LASSO: Least Absolute Shrinkage and Selection Operator
SGL: Sparse group LASSO
AUC: Area under the curve
OS: Overall survival
C-index: Concordance index
CNV: Copy number variation
DEGs: Differentially expressed genes
logFC: Log fold change
GSEA: Gene set enrichment analysis
GSVA: Gene set variation analysis
ssGSEA: Single-sample gene set enrichment analysis
FDR: False discovery rate
FPR: False positive rate
TP: True positive
TN: True negative
FP: False positive
FN: False negative
SP: Strongly positive
WP: Weakly positive
SN: Strongly negative
WN: Weakly negative
TIS: Tumor Inflammation Signature
EMT: Epithelial-mesenchymal transition
KM: Kaplan–Meier
GEO: Gene Expression Omnibus
TCGA: The Cancer Genome Atlas
GTEx: Genotype-Tissue Expression
NSCLC: Non-Small Cell Lung Cancer
LUAD: Lung Adenocarcinoma
LUSC: Lung Squamous Cell Carcinoma
LIHC: Liver Hepatocellular Carcinoma
COAD: Colon Adenocarcinoma
BRCA: Breast Invasive Carcinoma
HNSC: Head and Neck Squamous Cell Carcinoma
ROC: Receiver operating characteristic
OOF: Out-of-fold
CMS1–CMS4: Consensus molecular subtypes 1–4
