# Supplementary material for: Single-cell phenotype-associated subpopulation identification via transfer foundation model and statistical ensemble learning
Source: BMC Biol. 2026 Apr 29;24:140. doi: 10.1186/s12915-026-02613-8 (PMC13270573; doi:10.1186/s12915-026-02613-8)
Supplement: Supplementary file 1 — Additional file 1. Multi-Dimensional Comparison of scPASI and Existing Methods. [file 12915_2026_2613_MOESM1_ESM.docx]

**Table S1:** Multi-Dimensional Comparison of scPASI and Existing Methods

| Comparison Dimension | **scAB** | **Scissor** | **scIdentifier** | **scPASI** |
| --- | --- | --- | --- | --- |
| **Data preprocessing** | • Quality control and normalization using Seurat  • Highly variable gene selection  • Linear dimensionality reduction (PCA)  • Construction of cell–cell SNN graph and Laplacian matrix  • Computation of sample–cell correlation matrix | • Normalization of single-cell and bulk RNA-seq data  • Construction of cell–cell SNN graph and Laplacian matrix  • Computation of sample–cell correlation matrix | • Normalization of single-cell and bulk RNA-seq data  • Construction of gene–gene similarity graph (based on Pearson correlation of highly variable genes) and Laplacian matrix  • Use of expression matrix for regression-based risk score computation | • Extraction of cell embeddings using scFoundation model and Res-VAE  • Construction of cell–cell SNN graph and Laplacian matrix  • Initial cell grouping using Leiden algorithm  • Computation of sample–cell correlation matrix |
| **Core algorithmic framework** | Graph-regularized supervised non-negative matrix factorization (NMF) | Graph-constrained LASSO regression | Graph-constrained LASSO regression | Graph-constrained structured sparse regression (LASSO + Sparse Group LASSO) |
| **Modeling target (selected features)** | Cells | Cells | Genes | Cells |
| **Core output structure** | Cell loading matrix | Single vector of cell coefficients | Gene coefficient vector | Multiple cell coefficient vectors corresponding to different association strengths |
| **Cell–phenotype association measure** | Cell loadings within learned modes | Sign and magnitude of regression coefficients | Risk score | Stratified cell regression coefficients based on sign and magnitude (strong/weak associations) |
| **Output resolution** | Associated / non-associated modes | Positively / negatively associated cells | Positively / negatively associated cells | Strong positive / weak positive / strong negative / weak negative cells |
