# Supplementary material for: Single-cell phenotype-associated subpopulation identification via transfer foundation model and statistical ensemble learning
Source: BMC Biol. 2026 Apr 29;24:140. doi: 10.1186/s12915-026-02613-8 (PMC13270573; doi:10.1186/s12915-026-02613-8)
Supplement: Supplementary file 2 — Additional file 2. Stability Analysis of WP Cells Under Progressive Downsampling (COAD_tumors). [file 12915_2026_2613_MOESM2_ESM.docx]

**Table S2:** Stability Analysis of WP Cells Under Progressive Downsampling (COAD_tumors)

| Downsampling Ratio of Negative Cells | WP→SP | Flip Rate (%) |
| --- | --- | --- |
| 10% removed | 0 | 0.0000 |
| 20% removed | 0 | 0.0000 |
| 30% removed | 0 | 0.0000 |
| 40% removed | 1 | 0.1395 |
| 50% removed | 0 | 0.0000 |
| 60% removed | 0 | 0.0000 |
| 70% removed | 0 | 0.0000 |
| 80% removed | 0 | 0.0000 |
| 90% removed | 2 | 0.2789 |
