# Supplementary material for: Single-cell phenotype-associated subpopulation identification via transfer foundation model and statistical ensemble learning
Source: BMC Biol. 2026 Apr 29;24:140. doi: 10.1186/s12915-026-02613-8 (PMC13270573; doi:10.1186/s12915-026-02613-8)
Supplement: Supplementary file 3 — Additional file 3. Comparison of Leiden and Louvain Clustering Algorithms Across Resolutions on the Number of Correctly Identified Tumor Cells and AUC Values. [file 12915_2026_2613_MOESM3_ESM.docx]

**Table S3:** Comparison of Leiden and Louvain Clustering Algorithms Across Resolutions on the Number of Correctly Identified Tumor Cells and AUC Values

| Dataset | Cluster Algoritm | *resolution* | AUC | Number of Correctly Identified Tumor Cells |
| --- | --- | --- | --- | --- |
| NSCLC | Leiden | 0.1 | 0.9973113 | 435 |
|  |  | 0.2 | 0.9905 | 49 |
|  |  | 0.3 | 0.995 | 50 |
|  |  | 0.4 | 0.9913 | 171 |
|  |  | 0.5 | 0.9913289 | 173 |
|  |  | 0.6 | 0.9926 | 140 |
|  |  | 0.7 | 0.9922 | 170 |
|  |  | 0.8 | 0.9925 | 173 |
|  |  | 0.9 | 0.9922 | 168 |
|  | Louvain | 0.1 | 0.9973777 | 358 |
|  |  | 0.2 | 0.9973 | 435 |
|  |  | 0.3 | 0.9967 | 389 |
|  |  | 0.4 | 0.9906 | 49 |
|  |  | 0.5 | 0.9912 | 161 |
|  |  | 0.6 | 0.9912 | 161 |
|  |  | 0.7 | 0.9913 | 161 |
|  |  | 0.8 | 0.9926 | 138 |
|  |  | 0.9 | 0.9907 | 51 |
| COAD | Leiden | 0.1 | 0.9576805 | 171 |
|  |  | 0.2 | 0.9573575 | 169 |
|  |  | 0.3 | 0.9549884 | 163 |
|  |  | 0.4 | 0.9570882 | 451 |
|  |  | 0.5 | 0.9553115 | 390 |
|  |  | 0.6 | 0.9680719 | 446 |
|  |  | 0.7 | 0.9611802 | 167 |
|  |  | 0.8 | 0.9640338 | 175 |
|  |  | 0.9 | 0.9694718 | 18 |
|  | Louvain | 0.1 | 0.9576805 | 171 |
|  |  | 0.2 | 0.9576805 | 171 |
|  |  | 0.3 | 0.955796 | 399 |
|  |  | 0.4 | 0.9546654 | 163 |
|  |  | 0.5 | 0.9568729 | 439 |
|  |  | 0.6 | 0.9568729 | 439 |
|  |  | 0.7 | 0.9568729 | 439 |
|  |  | 0.8 | 0.9627955 | 168 |
|  |  | 0.9 | 0.9662413 | 167 |
