# Supplementary material for: Single-cell phenotype-associated subpopulation identification via transfer foundation model and statistical ensemble learning
Source: BMC Biol. 2026 Apr 29;24:140. doi: 10.1186/s12915-026-02613-8 (PMC13270573; doi:10.1186/s12915-026-02613-8)
Supplement: Supplementary file 4 — Additional file 4. Comparison of Four Regression Model Combinations on the Number of Correctly Identified Tumor Cells and AUC Values. [file 12915_2026_2613_MOESM4_ESM.docx]

**Table S4:** Comparison of Four Regression Model Combinations on the Number of Correctly Identified Tumor Cells and AUC Values

| Dataset | Regression Model Combination | Cluster Algorithm with *resolution* | AUC | Number of Correctly Identified Tumor Cells |
| --- | --- | --- | --- | --- |
| NSCLC | SGL_LASSO | Leiden0.5 | 0.9913289 | 173 |
|  |  | Louvain0.5 | 0.9912000 | 161 |
|  |  | Leiden0.6 | 0.9926000 | 140 |
|  |  | Louvain0.6 | 0.9912000 | 161 |
|  | SGL_Enet | Leiden0.5 | 0.9913289 | 176 |
|  |  | Louvain0.5 | 0.9912281 | 165 |
|  |  | Leiden0.6 | 0.9925724 | 143 |
|  |  | Louvain0.6 | 0.9911945 | 165 |
|  | SGL_3models | Leiden0.5 | 0.9962694 | 242 |
|  |  | Louvain0.5 | 0.9956308 | 229 |
|  |  | Leiden0.6 | 0.9854809 | 232 |
|  |  | Louvain0.6 | 0.9855146 | 172 |
|  | SGL_Scissor | Leiden0.5 | 0.9998320 | 328 |
|  |  | Louvain0.5 | 0.9998000 | 316 |
|  |  | Leiden0.6 | 0.9998000 | 296 |
|  |  | Louvain0.6 | 0.9998000 | 314 |
| COAD | SGL_LASSO | Leiden0.5 | 0.9553115 | 390 |
|  |  | Louvain0.5 | 0.9568729 | 439 |
|  |  | Leiden0.6 | 0.9680719 | 446 |
|  |  | Louvain0.6 | 0.9568729 | 439 |
|  | SGL_Enet | Leiden0.5 | 0.9553115 | 408 |
|  |  | Louvain0.5 | 0.9568729 | 456 |
|  |  | Leiden0.6 | 0.9680719 | 463 |
|  |  | Louvain0.6 | 0.9568729 | 456 |
|  | SGL_3models | Leiden0.5 | 0.9849244 | 495 |
|  |  | Louvain0.5 | 0.9849244 | 548 |
|  |  | Leiden0.6 | 0.9626878 | 546 |
|  |  | Louvain0.6 | 0.9621494 | 523 |
|  | SGL_Scissor | Leiden0.5 | 1 | 818 |
|  |  | Louvain0.5 | 1 | 848 |
|  |  | Leiden0.6 | 1 | 857 |
|  |  | Louvain0.6 | 1 | 828 |
