# Supplementary material for: Single-cell phenotype-associated subpopulation identification via transfer foundation model and statistical ensemble learning
Source: BMC Biol. 2026 Apr 29;24:140. doi: 10.1186/s12915-026-02613-8 (PMC13270573; doi:10.1186/s12915-026-02613-8)

**Figure S1:** Comparison of scPASI with and without PFM in terms of CNV scores for cells identified in the COAD dataset.


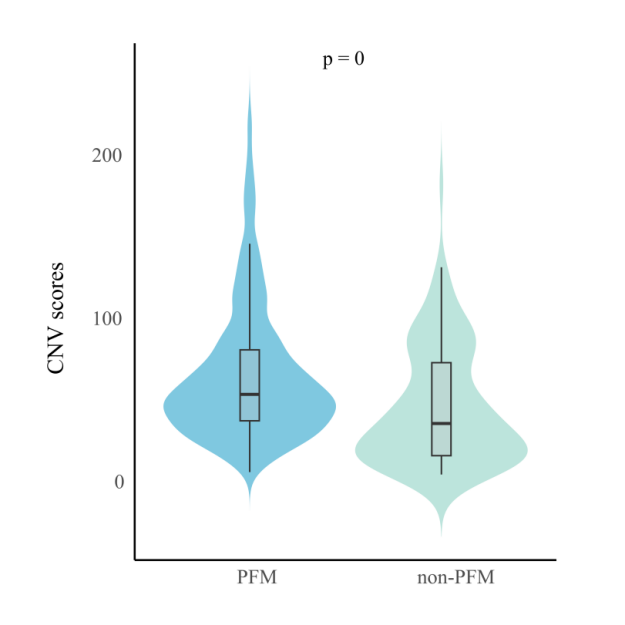

Supplement: Supplementary file 5 — Additional file 5. Comparison of scPASI with and without PFM in terms of CNV scores for cells identified in the COAD dataset. [file 12915_2026_2613_MOESM5_ESM.docx]
