# Supplementary material for: Single-cell phenotype-associated subpopulation identification via transfer foundation model and statistical ensemble learning
Source: BMC Biol. 2026 Apr 29;24:140. doi: 10.1186/s12915-026-02613-8 (PMC13270573; doi:10.1186/s12915-026-02613-8)

**Figure S2:** Expression of Tumor Malignancy Markers Across Identified Cell Subpopulations. (a) Expression of Gene S100A10. (b) Expression of Gene EMP2. (c) Expression of Gene RhoC.


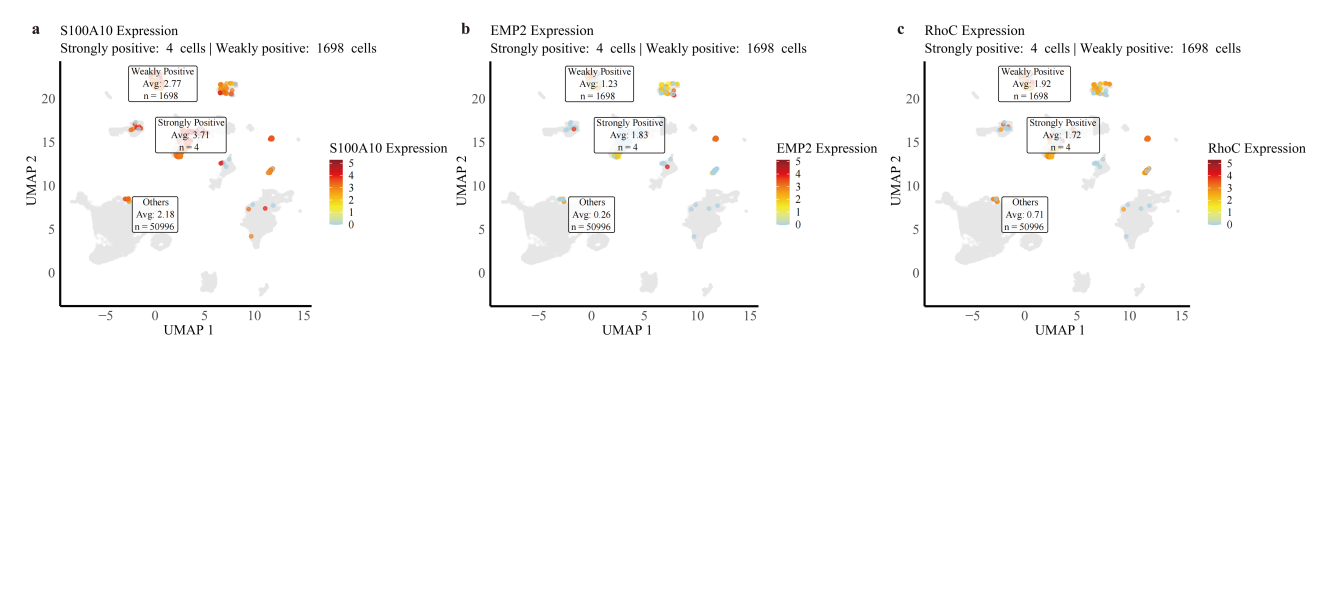

Supplement: Supplementary file 6 — Additional file 6. Expression of Tumor Malignancy Markers Across Identified Cell Subpopulations. (a) Expression of Gene S100A10. (b) Expression of Gene EMP2. (c) Expression of Gene RhoC. [file 12915_2026_2613_MOESM6_ESM.docx]
