# Supplementary material for: Single-cell phenotype-associated subpopulation identification via transfer foundation model and statistical ensemble learning
Source: BMC Biol. 2026 Apr 29;24:140. doi: 10.1186/s12915-026-02613-8 (PMC13270573; doi:10.1186/s12915-026-02613-8)

**Figure S3:** Expression of Immune-Related Markers Across Identified Cell Subpopulations. (a) Expression of Gene B2M. (b) Expression of Gene CCL5. (c) Expression of Gene CD3D.


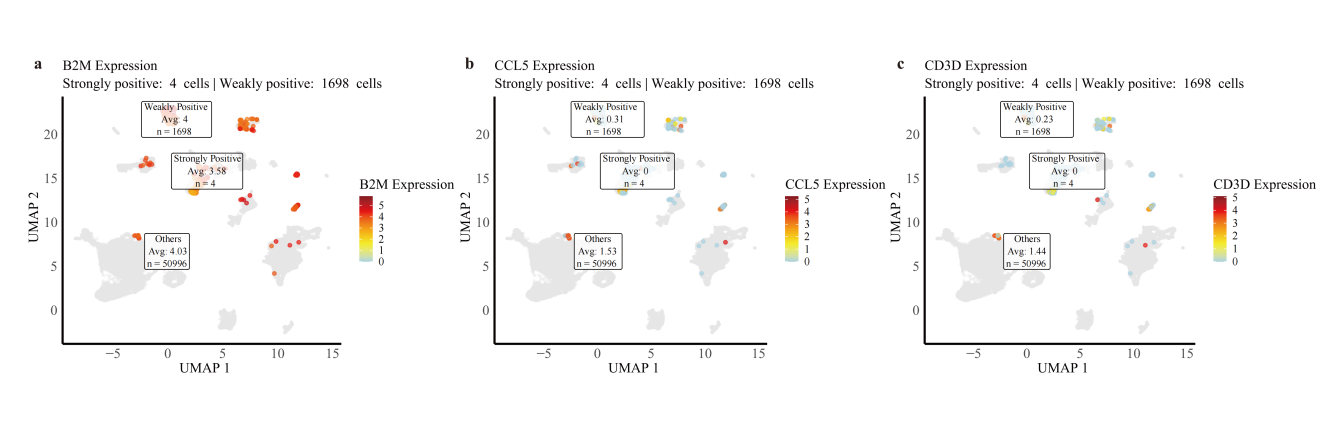

Supplement: Supplementary file 7 — Additional file 7. Expression of Immune-Related Markers Across Identified Cell Subpopulations. (a) Expression of Gene B2M. (b) Expression of Gene CCL5. (c) Expression of Gene CD3D. [file 12915_2026_2613_MOESM7_ESM.docx]
